# A recent review on ginseng’s effects on neurodegenerative diseases: active ingredients, mechanisms of action, applications, and delivery strategies

**DOI:** 10.3389/fnut.2026.1912520

**Published:** 2026-08-19

**Authors:** Li Sun, Xuelu Yu, Haoyuan Yin, Jinhao Bi, Hongqiang Lou, Hongyan Pei

**Affiliations:** 1 College of Medicine, Changchun Sci-Tech University, Changchun, China; 2 Naval Medical Center of People’s Liberation Army, Naval Medical University, Shanghai, China; 3 Neurovascular Surgery Clinical Center, The First Hospital of Jilin University, Changchun, Jilin, China; 4 Center for Infectious Disease Research, Westlake University, Hangzhou, Zhejiang, China; 5 School of Medicine, Jinhua University of Vocational Technology, Jinhua, China

**Keywords:** active ingredients, applications, delivery strategies, ginseng, mechanisms of action, neurodegenerative diseases

## Abstract

**Background:**

Neurodegenerative diseases (NDDs) pose a major health challenge due to their high prevalence and the lack of effective treatments; ginseng, as medicine and food homology, has potential neuroprotective effects.

**Methods:**

This article provides a systematic review of research conducted over the past five years on the use of ginseng to treat NDDs, summarizing and analyzing the findings in four key areas: active components, mechanisms of action, clinical applications, and novel delivery strategies.

**Results:**

Ginsenosides are the core active ingredients in ginseng, while polysaccharides, essential oils, and peptides also exert synergistic effects through various pathways; their mechanisms of action include regulating Aβ/tau protein aggregation, inhibiting microglial activation, reducing glutamate excitotoxicity, and restoring mitochondrial function and antioxidant balance. In models of AD, PD, HD, and ALS, ginseng’s active components have been shown to improve both behavioral and pathological indicators, while novel delivery strategies (nanoparticles, exosomes, and engineered cellular carriers) can significantly enhance blood–brain barrier permeability and brain-targeting efficiency.

**Conclusion:**

Ginseng exhibits protective effects against NDDs through multiple mechanisms of action. However, current evidence is largely limited to preclinical studies, and future efforts should focus on advancing the clinical translation of safe and effective brain-targeted delivery systems.

## 1 Introduction

Neurodegenerative diseases (NDDs), including Alzheimer’s disease (AD), Parkinson’s disease (PD), Huntington’s disease (HD), and amyotrophic lateral sclerosis (ALS), have become a major challenge that seriously threatens the health and quality of life of people worldwide (1). With the acceleration of population aging, the incidence and disease burden of these conditions continue to rise (2). However, currently available clinical treatments are extremely limited; most can only alleviate certain symptoms and are unable to effectively halt or reverse the progressive damage and death of neurons (3). Therefore, the search for multi-targeted, low-toxicity neuroprotective agents derived from natural products has become a research focus (4–6). *Panax ginseng* C. A. Mey. (Ginseng) has long been used to tonify the body, enhance cognitive function, and promote mental calmness (7). Modern research has confirmed that its various bioactive components exert significant protective effects on the central nervous system; however, systematic reviews on ginseng’s role in improving neurodegenerative diseases have been insufficient in recent years, particularly regarding its regulatory mechanisms within complex pathological processes and the progress in barrier-crossing delivery strategies.

**Figure 1:**
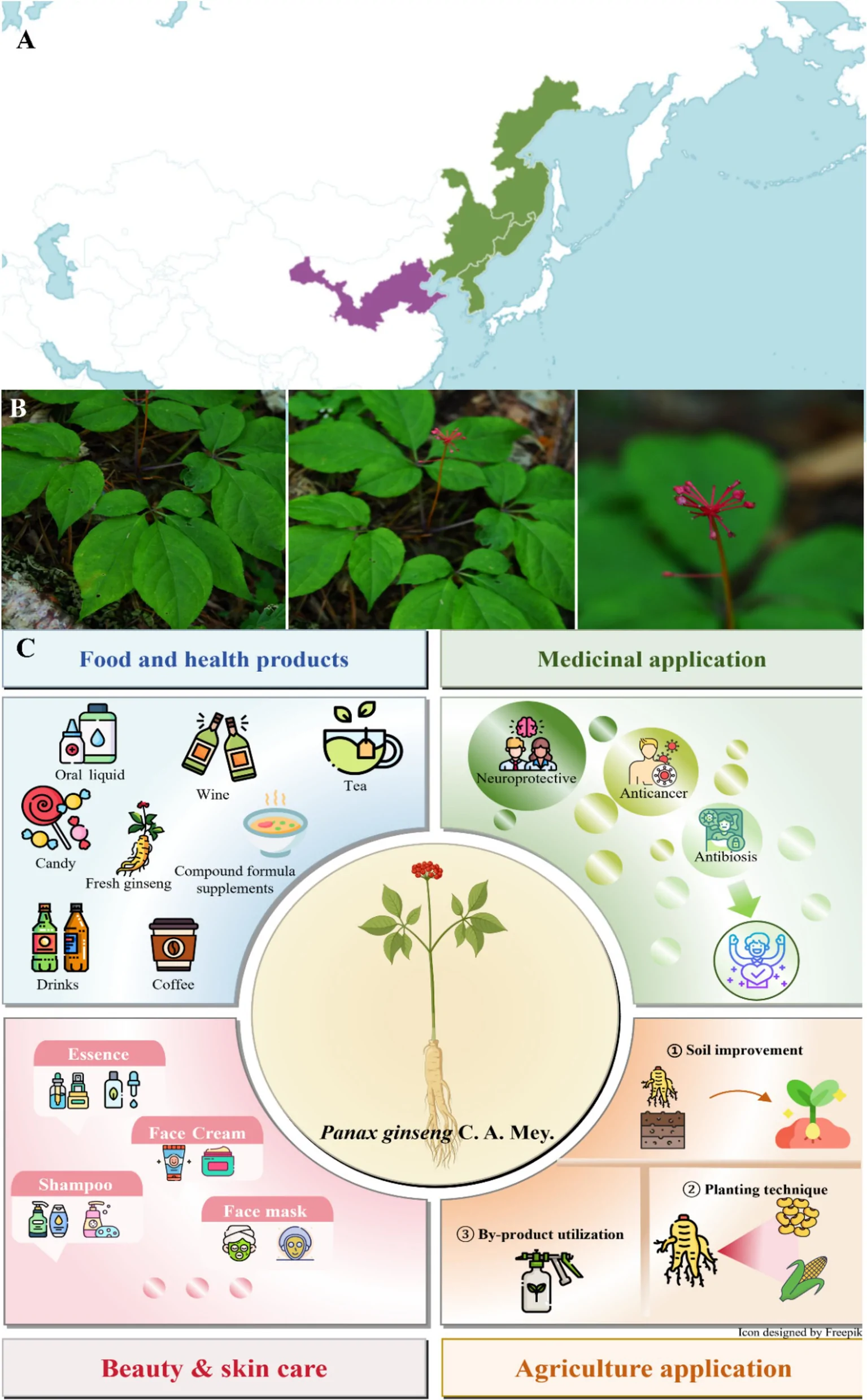
**(A)** Native (green) and introduced (purple) regions of ginseng. **(B)** The above-ground parts of the ginseng plant. **(C)** Ginseng has become deeply integrated into various sectors of the health industry.

*Panax ginseng* C. A. Mey. (Ginseng) are perennial herbs primarily found in Khabarovsk, Korea, Manchuria, Primorye, China North-Central (8). Ginseng was first documented in the Shennong Bencao Jing over 2,000 years ago. It is primarily distributed in the temperate forest regions of eastern Asia between 35° and 48° north latitude, with the Changbai Mountains region in Jilin, China, serving as the core production area (Figures 1A,B). To date, at least 200 different ingredients have been isolated from ginseng, including saponins, polysaccharides, essential oils, amino acids, peptides, flavonoids, and phenolic ingredients (9, 10). Among these ingredients, ginsenosides are considered the primary active constituents of ginseng, and most research has focused on this class of ingredients (11). The value of ginseng has long transcended traditional research; through modern technology and product innovation, it has become deeply integrated into various sectors of the health industry, including food and health products, medicinal applications, beauty and skincare, and agricultural applications (Figure 1C). However, it should be noted that the current evidence of potential benefits is primarily derived from animal studies and a small number of observational studies, and cannot yet be directly extrapolated to clinical patient populations.

The mechanisms by which ginseng improves NDDs include restoring protein homeostasis, alleviating neuroinflammation, inhibiting excitotoxicity, and correcting mitochondrial dysfunction and oxidative stress (12). However, the bioavailability of ginseng’s active components is generally low, and their ability to cross the blood–brain barrier is limited, which severely hampers their clinical translation (13). To overcome this challenge, researchers have recently developed novel delivery systems based on molecular strategies (nanoparticles, liposomes, exosomes, etc.), cellular strategies (engineered cell carriers, stem cell loading), and physical strategies, thereby enhancing the brain-targeting ability and therapeutic efficacy of ginseng’s active components (14). This review will systematically summarize the latest research progress on ginseng’s role in improving NDDs across four dimensions: active components, mechanisms of action, disease applications, and novel delivery strategies, with the aim of providing a reference for further exploration and clinical development in this field. Furthermore, although ginseng has demonstrated clear protective effects against NDDs through multi-target mechanisms, its clinical translation is limited by low brain exposure to its active ingredients; novel delivery strategies may offer a breakthrough.

## 2 Methods

**Figure 2:**
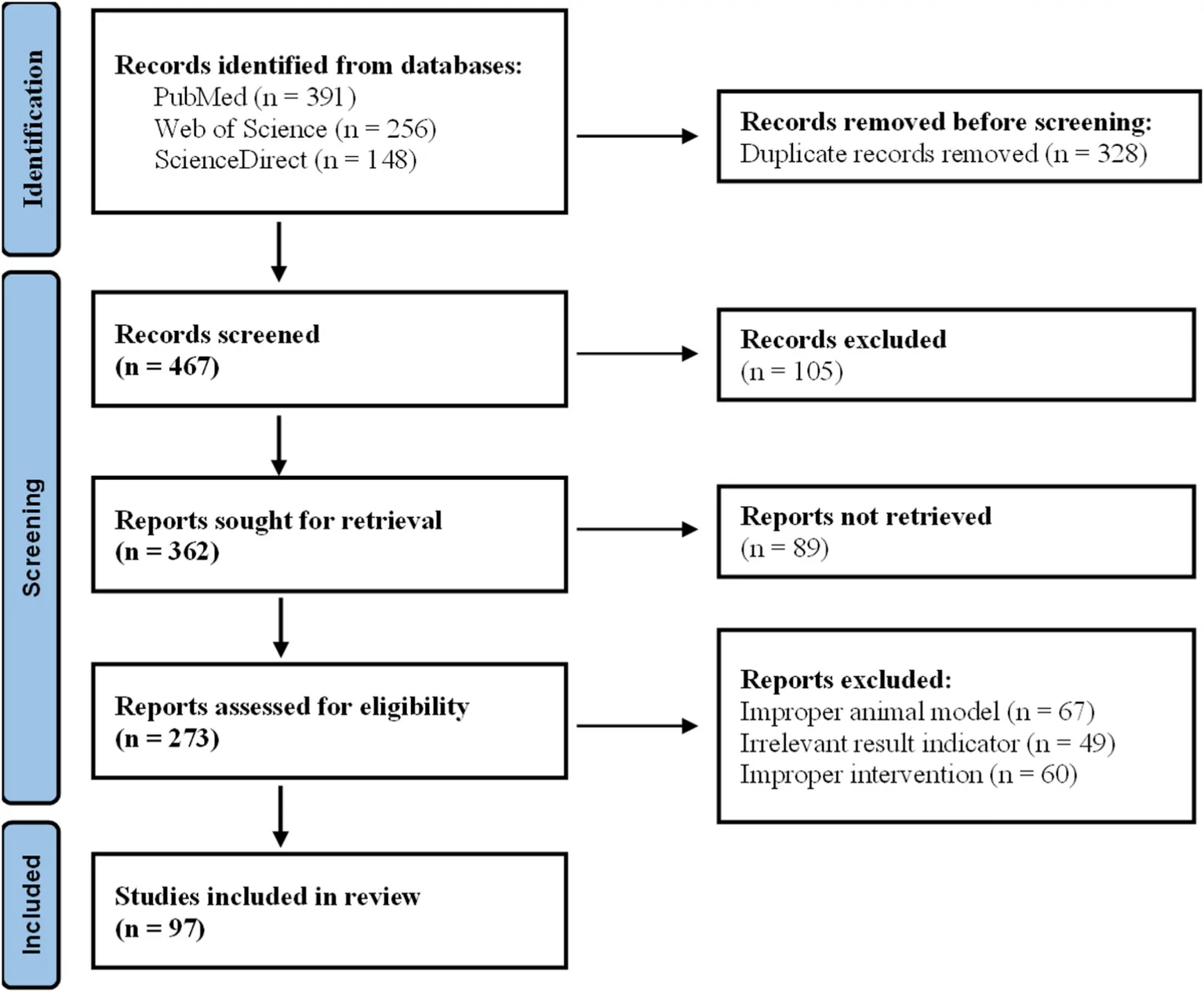
PRISMA flow diagram for literature screening.

This review was conducted through a systematic search of the PubMed, Web of Science, and ScienceDirect databases. It collected relevant literature published between January 2021 and June 2026 on the effects of ginseng and its active components in improving NDDs (primarily including Alzheimer’s disease, Parkinson’s disease, Huntington’s disease, and amyotrophic lateral sclerosis, among others). Search terms included “*Panax ginseng*,” “ginsenoside,” “chemical ingredient” “neurodegenerative diseases,” “NDDs” “Alzheimer’s disease,” “Parkinson’s disease,” “neuroprotection,” “drug delivery,” and “blood–brain barrier,” supplemented by manual searches of the reference lists of included studies to identify potential omissions. This study conducted literature screening in accordance with the PRISMA guidelines (Figure 2). First, relevant studies were identified through database searches. The identified studies were then scanned and preliminarily screened; after excluding duplicates and clearly irrelevant studies, the final set of included studies was obtained (*n* = 97). Inclusion criteria were: (1) the study was an original animal or cell experiment; (2) the intervention was ginseng or its active components; (3) the study subjects had neurodegenerative diseases such as Alzheimer’s disease, Parkinson’s disease, Huntington’s disease, or amyotrophic lateral sclerosis; (4) outcome measures included behavioral, pathological, or molecular biological parameters; (5) the language of the study was English. Exclusion criteria were: conference abstracts, case reports, duplicate publications, and studies with incomplete data. Two researchers independently screened the literature and extracted data; disagreements were resolved through discussion or third-party arbitration. We used the SYRCLE tool for assessing the risk of bias in animal studies to evaluate the methodological quality of the included animal studies and conducted corresponding risk-of-bias analyses for cell-based experiments based on their design characteristics. Therefore, this study is a narrative review.

## 3 Active ingredients in ginseng

Ginseng is a perennial herb of the genus in the family Araliaceae as one of the most important traditional Chinese medicine (15). It contains a variety of complex biologically active components, including ginsenosides, polysaccharides, volatile oils, amino acids, peptides, and others. Although numerous active ingredients have been isolated and identified, their oral bioavailability and ability to cross the blood–brain barrier are the two key rate-limiting factors determining their *in vivo* effects.

### 3.1 Saponin

**Figure 3:**
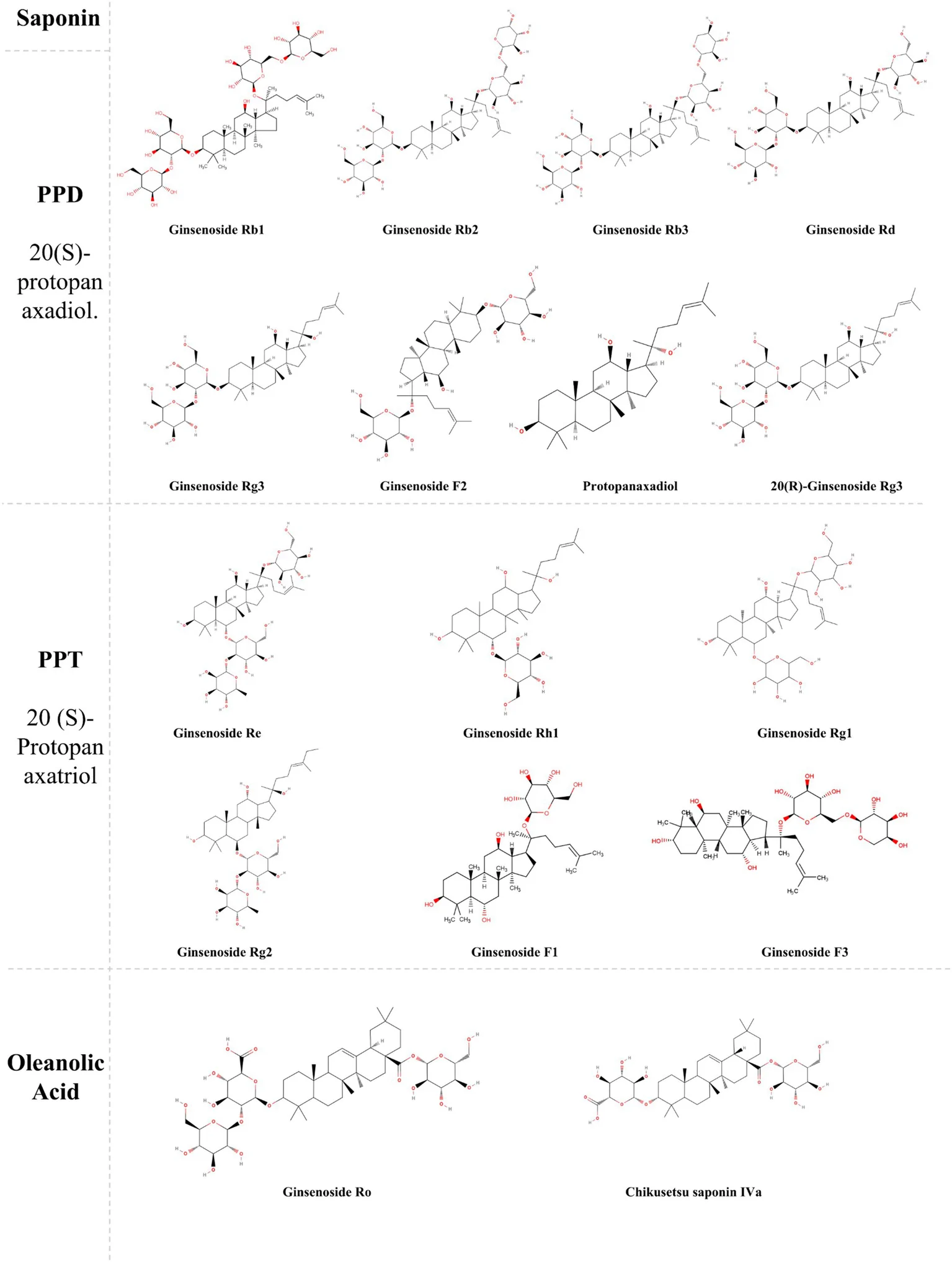
The structural formula of ginsenosides.

Saponins are complex organic ingredients widely found in plants, which are composed of saponin elements linked to sugars, and are a class of triterpenoids (16). Its structural basis is a combination of a tetracyclic steroidal structure and a sugar side chain. According to the different connection patterns between the steroidal nucleus and the sugar chain, as well as the structural variability of the sugar chain itself, ginsenosides can be classified into a number of different categories, including Rb1, Rb2, Rc, Rd., Re, Rf, Rg1, Rg2, and up to dozens of other different compounds (17). In addition, depending on the structure of the glycosides, they can be categorized into dammarane type, oleanane type and ocotillol type (18). Dammarane-type saponins include diol-type (PPD, A-type) and triol-type (PPT, B-type). Oleanolane type (C type) has oleanolic acid as the glycoside (Figure 3) (19).

### 3.2 Polysaccharides

Polysaccharides are complex polymeric glycopolymeric carbohydrates consisting of at least more than 10 monosaccharides linked by glycosidic bonds (20). As important pharmacodynamic active ingredients of ginseng, polysaccharides have received high attention. Ginseng polysaccharides are rich in monosaccharide chains such as L-arabinose (Ara), L-rhamnose (Rha), D-galactose (Gal), D-mannose (Man), D-galacturonic acid (GalA), D-glucuronic acid (GlcA), D-xylulose (Xyl) and D-galactose (Gal). In addition, ginseng polysaccharides are categorized into neutral and acidic polysaccharides based on whether they are charged or not. Neutral polysaccharides include dextran and arabinogalactan, while acidic polysaccharides include pectin-containing rhamnose and homogalacturonic acid (21, 22).

### 3.3 Volatile oil

Volatile oils are liquid mixtures of aromatic compounds extracted from plants, representing only a small fraction of total plant constituents. It consists of aldehydes, heterocycles, sesquiterpenes, fatty acids, fatty acid ester ingredients and alkanes. It has been shown that 369 metabolites in GVO, including 154 hydrocarbons, 35 ketones, 2 aldehydes, 55 esters, 37 alcohols, 12 acids, 22 nitrogen-containing metabolites, and 52 other metabolites, have been identified by modern techniques of GC–MS to assess the ingredient and quality of volatile oil (23). Many sesquiterpene hydrocarbons as well as oxygenated sesquiterpenes have also been identified as volatile constituents of ginseng. More than 15 sesquiterpenes have been identified as volatile components of ginseng (24).

### 3.4 Amino acids and peptides

In the 1980s, ginseng was found to have a rich concentration of amino acids, with more than 18 amino acids, 14 of which were highly accumulated (25). Some of these amino acids are essential amino acids that cannot be synthesized in human body, such as lysine, methionine, aspartic acid, phenylalanine, leucine, isoleucine, threonine and histidine. They are also used as important indicators for ginseng product quality control (26).

In addition, polypeptides also occupy a certain proportion in the ingredient of ginseng, and they have specific biological functions that ginseng’s original proteins and amino acids do not have. The research of ginseng peptides started in 1960s, German scientists extracted and isolated 4 peptides of amino acids from ginseng, and determined the molar ratio of amino acids of 2 ginseng peptides (27–29). Zhao et al. found that different parts of ginseng were rich in peptides, and there were significant differences in the peptide contents between ginseng main root and branch root, and between rehmanniae and baleen root (30).

### 3.5 Other ingredients

As a traditional precious Chinese herbal medicine, the study of ginseng’s pharmacological active ingredients has been extended from organic components such as saponins, polysaccharides and amino acids to the field of trace elements. It is found that ginseng contains more than 17 kinds of elements, including beneficial elements (e.g., germanium, tin, zinc, etc.) and harmful elements (e.g., arsenic, lead, mercury, etc.) (31). Polyacetylene is a group of secondary metabolites typically produced by members of the Umbelliferae, Araliaceae and Asteraceae families (32). Currently, more than 20 polyacetylene derivatives have been isolated from ginseng, including ginsenol, ginsenodiol and ginsenotriol. Recently, novel polyacetylene metabolites with carbonyl-substituted hydroxyl groups have been isolated, such as 9,10-epoxyheptane-4,6-diyne-3- 1, 1 -ethoxy-9,10-epoxyheptane-4,6-diyne-3- 1, and 9,10-epoxy-16-heptane-4,6-diyne-3- 1 (33). In addition, Ginseng contains vitamin B1, vitamin B2, vitamin B12, vitamin C, niacin, pantothenic acid, folic acid and other vitamins.

## 4 The mechanism of action of ginseng in the treatment of NDDs

**Figure 4:**
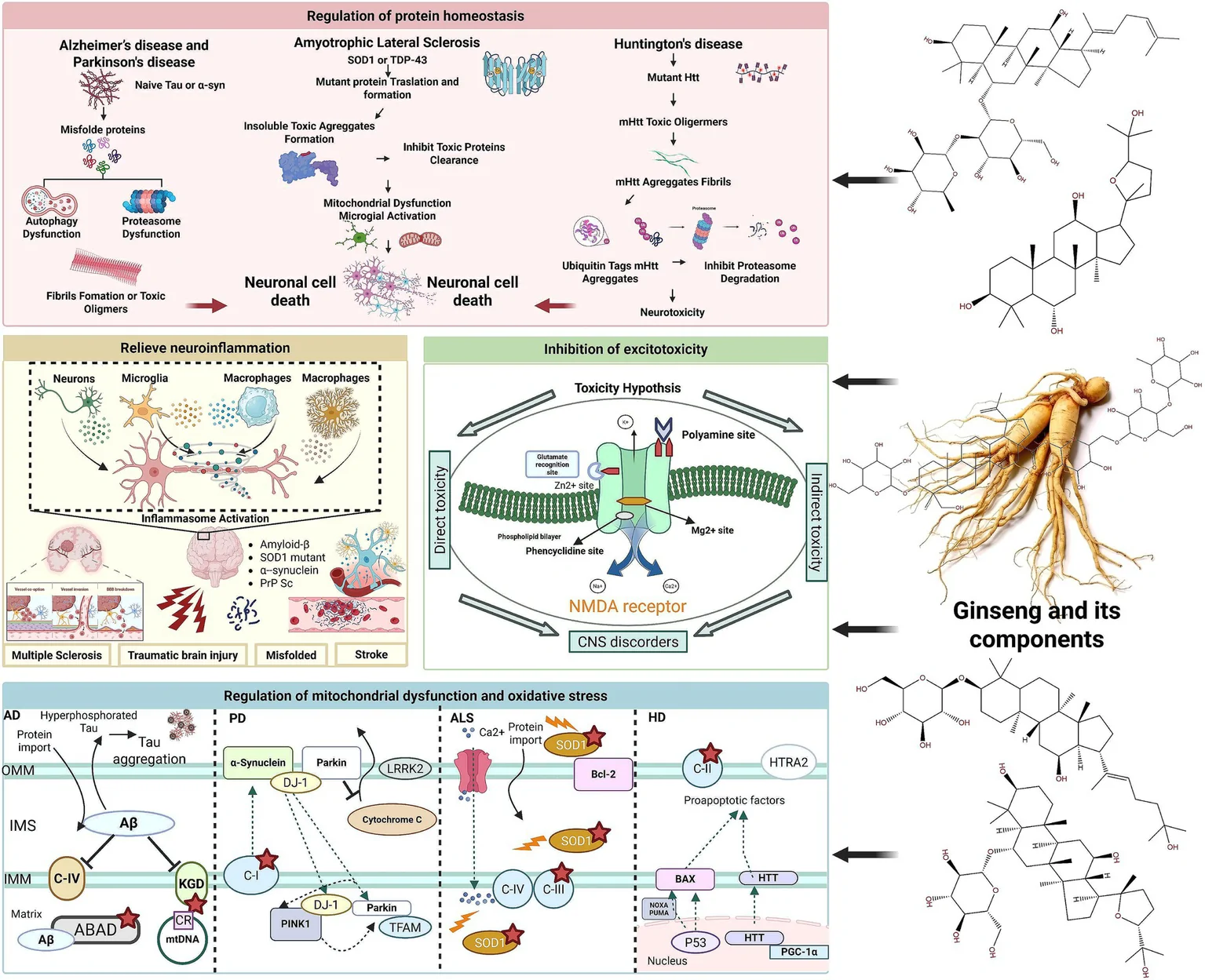
Ginseng and its active ingredients could regulate NDDs through various mechanisms. Image created with BioRender.com.

Ginseng and its active ingredients, such as saponins, regulate NDD diseases through a variety of mechanisms, including regulation of protein homeostasis, improvement of mitochondrial function, attenuation of oxidative stress, and inhibition of neuroinflammation and excitotoxicity (Figure 4). These mechanisms work together to provide a theoretical basis for the application of ginseng in the treatment of NDDs.

### 4.1 Regulation of protein homeostasis

One of the representative pathological features of NDDs is the imbalance in protein homeostasis induced by misfolding, leading to abnormal aggregation of toxic proteins and loss of neuronal function (34). In recent years, researchers have increasingly explored the complex link between protein aggregation and NDDs, with a particular focus on four major proteins, Aβ and tau proteins in AD, *α*-synuclein (α-Syn) in PD, TDP-43 in ALS, and mutant Htt in HD, all of which form pathologic aggregates due to misfolding or aberrant post-translational modifications (35). In addition, biometallic ions Fe^2+^, Cu^2+^, and Zn^2+^ are involved in the regulation of enzyme activities and redox homeostasis at physiological concentrations, but their aberrant accumulation can exacerbate the progression of NDDs through multiple mechanisms (36).

Previous studies have found that certain ginsenosides can reduce Aβ levels in primary cultured neurons and in the brains of AD model mice. However, the specific mechanisms underlying this effect remain unclear. One study investigated the relationship between capacitive Ca^2+^ entry (CCE) and Aβ production by examining the effects of various ginsenosides on CCE levels. The results showed that ginsenosides capable of reducing Aβ (such as Rk1, Rg5, and Rg3) enhanced CCE. However, ginsenosides lacking Aβ-lowering activity (such as Re and Rb2) failed to enhance CCE. Furthermore, the enhancing effect of ginsenosides on CCE could be blocked by the CCE inhibitor 2-aminoethoxydiphenylboronate (2APB) (37). Xu et al. found that water extract of ginseng (WEG) protects neurons in the brains of mice with Parkinson’s disease by inhibiting the aggregation of *α*-Syn in the brain and increasing the expression levels of tyrosine hydroxylase (TH) in the brain (38). Another study found that ginsenoside Re (G-Re) alleviates motor impairments in a mouse model of Parkinson’s disease, reduces damage to dopaminergic neurons and *α*-Syn aggregation, and enhances antioxidant capacity (39). Similarly, researchers have found that forest-grown ginseng (GSF) promotes PC12 cell proliferation, reduces IL-8 levels, increases SOD activity, and alleviates Aβ25–35-induced inflammation and oxidative stress. This is primarily because GSF alleviates AD by reducing the deposition of Aβ protein in mouse brain tissue (40). In addition, intraperitoneal injection of Rg1 (10 mg/kg/d) for 30 d in a 5XFAD mouse model activated the PINK1/Parkin pathway to restore mitochondrial autophagy and induced phagocytosis in microglia to accelerate the reduction of Aβ deposition in the hippocampus of AD mice (41). In a study on the neuroprotective effects of ginsenoside Rb1 in the cerebral cortex of adult male albino mice treated with AlCl3, the administration of 70 mg/kg/d of ginsenoside Rb1 for 8 consecutive weeks showed potential neuroprotective effects of inhibiting the formation of Aβ and phosphorylated tau proteins, correcting oxidative stress, anti-apoptotic effects, and reducing glial cell formation (42). The aforementioned effects on protein aggregation have so far been observed only in cellular models or rodents; there is currently no direct evidence regarding the actual efficiency of Aβ and *α*-Syn clearance in the human brain.

### 4.2 Relieve neuroinflammation

One of the hallmarks of neurodegenerative events is an impaired inflammatory response (43). Neuroinflammation has been observed to be both a pathologic outcome and a driver of disease progression in AD, PD, ALS, and HD (44). Although the core triggers are different for each disease, they all contribute to a vicious cycle of neuronal injury by activating microglia and astrocytes and releasing pro-inflammatory factors and oxidative stress products (45). A*β* oligomers trigger the NF-κB signaling pathway to release pro-inflammatory factors such as IL-1β, IL-6, and TNF-*α* by binding to pattern recognition receptors on the surface of microglia (e.g., TLR4, CD36, and TREM2) (46–49). Neurons release α-syn-containing exosomes that activate neighboring astrocytes and amplify inflammatory signals (50). Mutant SOD1 activates the NF-κB pathway in microglia and astrocytes to release IL-6 and TNF-α (51). mHTT upregulates IL-1β and TNF-*α* expression by activating the IKK/NF-κB pathway in microglia (52). mHTT upregulates IL-1β and TNF-α expression by activating the IKK/NF-κB pathway in microglia (53).

For example, a study analyzing existing research and network pharmacology findings found that the neuroprotective effects of ginsenosides are primarily mediated through anti-inflammatory, anti-apoptotic, and antioxidant stress mechanisms, and may be associated with the PI3K/Akt, BDNF/TrkB, MAPKs, NF-κB, Nrf2, and Wnt/β-catenin signaling pathways (54). Ginsenoside Ro (Ro) significantly improves spatial exploration ability, reduces anxiety, and alleviates memory and cognitive impairments in AD model mice. This is primarily because Ro inhibits neuronal apoptosis (by downregulating Bax and Caspase-3 and upregulating Bcl-2), reduces neuroinflammation (by decreasing the activation of IBA1^+^ microglia and GFAP^+^ astrocytes, reducing pro-inflammatory factors TNF-α, IL-1β, and IL-6, and increasing the anti-inflammatory factor IL-10), and suppresses the phosphorylation levels of p38 and JNK in the MAPK signaling pathway (55). Similarly, ginsenoside Rg2 (GRg2) enhances learning, memory, and cognitive function. In addition, GRg2 effectively suppresses the growth of Bacteroides and *Helicobacter pylori*, and reduces the levels of acetone and trimethylamine N-oxide. These metabolites have been found to be closely associated with neuroinflammation. GRg2 effectively inhibits the activation of astrocytes and microglia in the brains of APP/PS1 mice and reduces the expression of neuroinflammatory mediators IL-6, IL-1β, and TNF-*α* (56). In addition, Ginsenoside Re can inhibit Aβ₁-₄₂-induced NLRP3 inflammasome activation by reducing mitochondrial reactive oxygen species (mtROS), maintaining mitochondrial membrane potential, and activating autophagy via the SIRT1/AMPK/mTOR signaling pathway (57). Another study found that purified panaxadiol saponin fraction (PDSF) almost completely blocked rotenone (ROT)-induced p65 activation and maintained anti-inflammatory and pro-inflammatory factors at normal levels, preserving BBB integrity; however, purified panaxatriol saponin fraction (PTSF) exacerbated p65 activation, leading to BBB damage. PDSF exerts a significant protective effect against ROT-induced PD in rats by protecting cerebrovascular endothelial cells (CECs), glial cells, and dopaminergic neurons (DNs), likely by inhibiting NF-κB p65-mediated neuroinflammation in CECs and directly protecting glial cells and neurons from ROT-induced toxicity (58). The anti-inflammatory effects of ginseng’s active components are highly dependent on the inhibition of the NLRP3 inflammasome–IL-1β pathway, whereas interventions that merely reduce TNF-α or IL-6 levels are often insufficient to reverse cognitive or motor impairments in animal models. This suggests that targeting specific inflammatory pathways rather than relying on broad-spectrum anti-inflammatory approaches is a more effective strategy.

### 4.3 Inhibition of excitotoxicity

Excitotoxicity is a pathological process caused by excessive activation of postsynaptic membrane NMDA receptors by excitatory neurotransmitters such as glutamate, leading to intracellular Ca^2+^ overload, mitochondrial damage, free radical generation, and apoptosis (59, 60). Specifically, PD-focused AMPA receptor-dopaminergic loop imbalance and hyperactivation of NMDA receptors (especially NR2B subunit-containing types) are central to AD excitotoxicity (61, 62). HD is driven by an NR2B-mHTT-mitochondrial interaction. mHTT directly binds the NR2B subunit and enhances NMDA receptor current strength in striatal medium-sized multispin neurons (MSNs). At the same time, mHTT inhibits mitochondrial calcium buffering capacity and amplifies Ca^2+^ signaling toxicity. Specific pathology of chronic NDDs (63–65).

A review discusses the active components of ginseng that improve AD and their applications in spatial metabolomics. The article notes that glutamate (Glu) and aspartate (Asp) are the primary excitatory neurotransmitters in the hippocampus and cerebral cortex, and excitotoxicity is one of the key pathological mechanisms of AD. Glutamate-treated cells exacerbate Aβ-induced neurotoxicity, whereas active components of ginseng can mitigate this excitotoxic damage by modulating the glutamatergic system (66). Similarly, some studies have highlighted the potential of ginseng and its bioactive components in the treatment of Alzheimer’s disease. These studies suggest that ginsenosides can mitigate excitotoxicity by modulating glutamatergic neurotransmission and inhibiting the excessive activation of NMDA receptors (67). For example, ginsenoside Rg3 protects neurons from NMDA-induced death by competitively interacting with the glycine binding site of the NMDA receptor. Another study reported that ginsenoside Rk1 effectively inhibits NMDA receptors by interacting with the polyamine-binding site of the NMDA receptor channel complex. Furthermore, ginsenoside Rd. can inhibit the excessive phosphorylation of the NR2B subunit *in vivo* and reduce its expression levels (68). An *in vivo* study found that ginsenoside Re (GRe) significantly attenuated kainate (KA)-induced seizures and excitotoxicity in the hippocampus of mice (69). The researchers investigated whether GRe could modulate mitochondrial dysfunction induced by the L-type calcium channel activator Bayk-8644. The GRe-mediated inhibition of PKCδ and its neuroprotective effects were comparable to those of the ROS inhibitor N-acetylcysteine, the mitochondrial protector cyclosporine A, the microglial inhibitor minocycline, or the PKCδ inhibitor rotolin (70).

### 4.4 Regulation of mitochondrial dysfunction and oxidative stress

Mitochondrial dysfunction, as the energy factory and redox regulatory center of the cell, plays an important role in NDDs (71). In AD, ROS production and inhibition of energy metabolism induce enhanced levels of Aβ in cells, which can alter mitochondrial function (72). Aβ affects complex IV (C-IV) (73) and *α*-ketoglutarate dehydrogenase (KGD) activity (74) and binds to Aβ-binding alcohol dehydrogenase (ABAD). Amyloid precursor protein (APP) also impairs mitochondrial protein inputs and increases the proteolytic activity of high-temperature demand protein A2 (HTRA2) (75), leading to cleavage of IAPs (inhibitors of apoptosis) and cell death. Among the mitochondrial defects in PD, complex I dysfunction has a central place (76). Complex I (C-I) activity is reduced in PD (77). Mutations in the mtDNA-encoded complex I subunit, 12SrRNA, and polymerase *γ* (POLG) also contribute to Parkinson’s syndrome. A number of proteins, including the Lewy body-associated protein α-Syn, protein deglycosylase (DJ-1), parkin, and kinase (LRRK2), are partially or completely localized in the mitochondria and are associated with PD. Physical associations have been reported between DJ-1 and α-Syn, DJ-1 and parkin, and DJ-1 and PINK1. There is also genetic evidence that DJ-1, PINK1 and parkin function sequentially in the same pathway (78). Increased expression of mutant SOD1 in ALS alters ETC activity and reduces mitochondrial calcium loading capacity. Mutant SOD1 promotes aberrant mitochondrial ROS production and the formation of aggregates that may block OMM protein import mechanisms or bind and sequester the anti-apoptotic protein Bcl-2 (79). Complex II activity is reduced in HD brains (80). Overexpression of the complex II subunit reduces cell death in striatal neurons expressing mutant HTT (81). Mutant HTT also increases sensitivity to calcium-induced cytochrome c release or translocation to the nucleus to affect expression of genes associated with apoptosis (82).

A study investigated the regulatory effects of ginsenosides Rg1 and Rg2 on autophagy and oxidative stress in neuroblastoma cells overexpressing Aβ(1–42). The results showed that Rg1 and Rg2 can upregulate autophagy levels in neuronal cells, as evidenced by increased expression of LC3II and Beclin-1 proteins, as well as decreased p62 levels (83). Similarly, ginsenoside Rg1 (Rg1) exerts its anti-AD effects by inhibiting neuronal autophagy through the suppression of FGR expression. Quan et al. demonstrated, using Aβ₁₋₄₂-induced AD mouse models and HT22 cell models, that FGR expression is significantly elevated in the hippocampus under AD conditions. Rg1 intervention downregulates FGR levels, thereby inhibiting hyperactivated autophagy (by decreasing the LC3 II/I ratio and increasing p62), and increases SIRT1 expression while decreasing BACE1 levels, thus reducing Aβ production and restoring autophagy homeostasis (84). These two studies are not contradictory; rather, they reflect Rg1’s bidirectional regulatory role on autophagy levels under different pathological conditions. Specifically, it is activated when autophagy is insufficient and inhibited when autophagy is excessive, with the ultimate goal of restoring autophagy homeostasis. In an *in vitro* experiment, G-Re was found to inhibit Aβ1-42-induced activation of the NLRP3 inflammasome. Mechanistically, G-Re inhibits inflammasome activation by reducing mitochondrial reactive oxygen species (mtROS) levels, maintaining mitochondrial membrane potential, and activating autophagy through the SIRT1/AMPK/mTOR signaling pathway (57). In addition, treatment with 20-(R)-protopanaxadiol (R-PPD) and 20-(S)-protopanaxadiol (S-PPD) alleviated MPTP-induced behavioral deficits. Furthermore, these ingredients protected against MPTP-induced neuronal damage and mitochondrial dysfunction, and reduced the abnormal expression of Cyt C, Bax, caspase-3, and Bcl-2 (85). Zhao et al. found that ginsenoside SumI effectively lowered lysosomal pH, promoted autophagosome formation, increased autophagy flux, and facilitated the transport of misfolded proteins to lysosomes for degradation in *Caenorhabditis elegans* (86). It is worth noting that the antioxidant effects of most ginseng components are more likely to be downstream consequences of upstream regulatory mechanisms—such as enhanced autophagy and inflammation suppression—rather than primary targets of action. Future research should not treat antioxidant activity as an isolated screening criterion (Table 1).

**Table 1:** The core pathogenesis of AD, PD, ALS, and HD.

Core pathogenesis	AD	PD	ALS	HD
Protein homeostasis imbalance and abnormal aggregation	Aβ plaques coexist with tau neurogenic fiber tangles (NFTs), where Aβ promotes the spread of tau pathology and tau inhibits neuronal activity, which together lead to cognitive decline	Lewy vesicles of α-Syn predominantly affect nigral dopaminergic neurons, but conformational polymorphisms may lead to different isoforms	Cytoplasmic aggregation of TDP-43 interferes with RNA metabolism and disrupts axonal transport, leading to motor neuron degeneration	Transcriptional interference with mHTT
Mitochondrial dysfunction and oxidative stress	Aβ oligomers directly embedded in the inner mitochondrial membrane inhibit complex IV activity and induce a mitochondrial ROS burst; hyperphosphorylated tau proteins translocate to the mitochondria, blocking ETC electron transfer and amplifying oxidative damage	Mutant α-Syn accumulates at the inner mitochondrial membrane and inhibits complex I function while activating the MAO-B enzyme to promote dopamine metabolism to produce reactive quinones; Mutations in the Parkin gene lead to impaired clearance of damaged mitochondria and increased accumulation of mitochondrial debris in the substantia nigra region	Mutant SOD1 loses its antioxidant function and accumulates abnormally in the mitochondrial cristae lumen, inducing mitochondrial calcium overload and ROS generation; TDP-43 cytoplasmic aggregation inhibits mitochondrial mRNA translation, leading to reduced respiratory chain protein synthesis	PGC-1α expression is downregulated and GABAergic neuron function is impaired
Neuroinflammation	CNS-resident microglia population driven, activation of NLRP3 inflammatory vesicles, activation of AIM2 inflammatory vesicles	α-Syn is shown to trigger activation of NLRP3 inflammatory vesicles in human monocytes and BV2 microglia nigral-specific inflammation	SOD1 mutants activate microglia in a TLR-dependent manner to promote pro-inflammatory IL-1β and TNFα production	mHTT upregulates IL-1β and TNF-α expression by activating the IKK/NF-κB pathway in microglia; binds to NLRP3 inflammatory vesicles, promotes IL-18 secretion, and accelerates striatal neuronal death
Excitotoxicity	NMDA receptor overactivation, calcium-dependent tau pathology, synaptic loss	Loss of dopaminergic synapses and imbalance of striatal loops	Excessive striatal glutamatergic input and neuronal calcium overload	Motor neuron glutamate excitotoxicity

## 5 Research on the application of ginseng in NDDs

### 5.1 Alzheimer’s disease

AD is a common form of dementia characterized by pathological deposits of beta-amyloid plaques and tau tangles, leading to progressive memory loss and cognitive decline (87). A study using *in vitro* and *in vivo* experiments found that administration of PEGylated ginsenoside Rg3 (GRg3)-loaded niosomes or GRg3 significantly reduced total antioxidant capacity, malondialdehyde (MDA) levels, and the gene expression of caspase-3. *In vivo* evaluations showed that GRg3 administration reduced neurovascular damage and preserved the integrity of the blood–brain barrier (BBB) and the hippocampus (88). Using BV2 microglia and HT22 neurons as in vitro models, Xie et al. found that pretreatment with ginsenoside C-K (GCK) significantly improved Aβ₁₋₄₂ oligomer-induced dysfunction in BV2 cells, suppressed the secretion of pro-inflammatory cytokines, and inhibited the activation of the NF-κB pathway (89). *In vivo* studies have shown that Rg1 treatment significantly improves cognitive dysfunction in APP/PS1 transgenic mice. Mechanistic studies revealed that Rg1 activates AMPK, enhances p-AMPK expression, and simultaneously inhibits Drp1. It upregulates the expression of mitochondrial fusion-related proteins OPA1, Mfn1, and Mfn2, thereby inhibiting excessive mitochondrial fission and promoting mitochondrial fusion (90). Fang and colleagues found that Rg1 enhances the viability and induces autophagy in Aβ1-42-treated BV2 cells, while also inhibiting apoptosis and inflammation. Rg1 can suppress microglial apoptosis and inflammation by regulating the GATA4/PDE4A/PI3K/AKT axis, thereby mitigating the progression of AD (91). Similarly, ginsenoside Rg2 (G-Rg2) exerts a protective effect against AD and improves the integrity of the blood–brain barrier (BBB). Specifically, in mouse astrocytes (MA-c), G-Rg2 similarly inhibited the activation of oxidative stress and Toll-like receptor pathways. Further in vitro studies demonstrated that G-Rg2 can suppress the activation of MA-c, thereby reducing the degradation of tight junctions (TJs) in bEnd3 cells and enhancing the integrity of the BBB (92). Yin and his colleagues found that Rg2 exerts its anti-AD effects by downregulating the expression of three proteins—LGMN, LAMP1, and PSAP—in the lysosomal pathway (93). Furthermore, treatment with total ginsenosides (TG) improved spatial learning and memory in APP/PS1 mice while reducing Aβ accumulation in the cortex and hippocampus. In N2a/APP695 cells, TG treatment attenuated the secretion of Aβ_1–40_ and Aβ_1–42_—which act as PPARγ agonists—by inhibiting the translocation of NF-κB p65. Furthermore, TG treatment reduced the expression of BACE1, PS1, and PS2, genes associated with the amyloid precursor protein pathway (94). Quan et al. found that ginsenoside Rg1 can inhibit apoptosis in primary cultured hippocampal neurons induced by Aβ 1–42 treatment, reduce intracellular and extracellular levels of Aβ 1–42, and inhibit the translocation of ERK from the cytoplasm to the nucleus (95). *In vitro* experiments have shown that RK3 can prevent Aβ-induced damage in primary cultured neurons and promote the proliferation of PC12 cells as well as the expression of synapse-related proteins (96).

### 5.2 Parkinson’s disease

PD clinically manifests as motor disorders such as resting tremor, rigidity, and bradykinesia. A report indicates that ginsenosides exert neuroprotective effects in NDDs by upregulating BDNF and activating the BDNF/TrkB signaling pathway. Researchers hypothesize that ginsenosides may protect neurons by activating the BDNF/TrkB pathway, thereby improving Parkinson’s disease (97). Ginseng protein (GP) significantly improves behavioral deficits in PD mice. GP significantly improved the distribution of cells in the hippocampus and the structure of the striatum in these mice, reduced *α*-Syn aggregation, and increased the expression of tyrosine hydroxylase (TH) in the brain. Analysis of the gut microbiota indicated that GP partially restored the disrupted gut microbiota balance in mice with chronic Parkinson’s disease (98). Similarly, CK demonstrated significant therapeutic effects in PD mice, improving behavioral abnormalities such as spatial memory and motor coordination. It increased the activity of T-AOC and GSH-Px, reduced MDA levels, thereby alleviating oxidative stress damage, inhibited apoptosis of dopaminergic neurons in the substantia nigra of PD mice, increased TH expression, and prevented the aggregation of α-Syn in the substantia nigra. Microbiome analysis indicates that CK treatment can reshape the gut microbiota of PD mice by increasing the abundance of beneficial bacteria (*Bacteroides*) and reducing the number of pathogenic bacteria (*Actinobacteria*) (99). Other studies have reported that ginsenoside Rk3 (Rk3) slows the progression of Parkinson’s disease (PD) by modulating the gut microbiome, highlighting its therapeutic potential for NDDs mediated by the gut-brain axis. Zhou and his colleagues found that Rk3 significantly increased levels of the microbial metabolite butyrate, which protects dopaminergic neurons and alleviates neuroinflammation by inhibiting histone deacetylase (HDAC) activity and activating the JAK/STAT3 signaling pathway (100). Reviews have reported that Rg1 may protect neurons and improve movement and cognitive impairments associated with PD through various mechanisms. However, the clinical application of Rg1 faces challenges in current studies, such as low bioavailability and a lack of comprehensive long-term safety and efficacy data (101). Wang et al. found that Rg1 treatment significantly alleviated both motor and non-motor symptoms associated with Parkinson’s disease. Under chronic stress conditions, Rg1 treatment selectively reduced the levels of the stress-sensitive protein RTP801 in the substantia nigra pars compacta (SNc) without affecting the acute stress response. HPLC-MS/MS analysis combined with site-specific mutagenesis revealed that Rg1 promoted the ubiquitination and subsequent degradation of RTP801 at residues K188 and K218, a process mediated by the Parkin RING2 domain (102). Furthermore, ginsenoside Re (Re) has demonstrated consistent neuroprotective effects in both human induced pluripotent stem cell-derived dopaminergic neurons and a Drosophila model of Parkinson’s disease. This is primarily manifested by Re directly binding to Drp1 across multiple species via the highly conserved L94 residue, triggering robust S616 phosphorylation, promoting the translocation of Drp1 to mitochondria, and thereby restoring the fission-fusion balance. Through the Drp1-Atg1/ULK1 axis, spatio-temporally coupled fission-fusion of mitochondria is further facilitated, leading to the initiation of autophagy and ensuring the effective clearance of damaged organelles (103). A *in vivo* study found that Rg3 effectively protects dopaminergic neurons and improves motor dysfunction (104). Currently, all reported protective effects related to PD have been observed in chemically induced models—such as those using MPTP and rotenone—and in transgenic mice; there are no published randomized controlled trials (RCTs) confirming that the active components of ginseng have a definitive beneficial effect on motor symptoms in patients with PD.

### 5.3 Huntington’s disease

Huntington’s disease (HD) is an autosomal dominant disorder caused by an abnormal expansion of CAG repeats in the huntingtin gene. It is characterized by the selective loss of medium spiny neurons in the striatum and manifests as choreiform involuntary movements, as well as cognitive and psychiatric symptoms. Lee et al. noted that ginsenosides Rg3 and Rf have the potential to reduce mutant huntingtin (mHtt) aggregation and apoptosis, making them promising candidates for treating the Huntington’s disease phenotype. In HD cell models, treatment with ginsenosides Rg3 and Rf reduced the number of mHtt-aggregate-positive cells and decreased lysosomal content. Molecules associated with mitochondrial biogenesis, such as PGC-1α and phosphorylated CREB, were upregulated, while apoptotic molecules p53, Bax, and cleaved caspase-3 were downregulated (105). In recent studies, researchers have used computational methods to investigate the therapeutic potential of ginseng for early-stage HD. Molecular docking calculations revealed that chemical components in ginseng, such as schizandrin (−10.530), ergosterol (−10.124), and protopanaxadiol (−9.650), exhibit higher binding affinities than the native ligand (−7.748), and these ingredients were identified as potential antagonists of the dopamine D1 receptor. The good geometric and thermodynamic stability of the schizandrin, protopanaxadiol, and panaxoside complexes suggests that these three molecules may inhibit the normal function or excessive activation of the receptor (106). A *in vivo* study using the R6/2 transgenic mouse model of Huntington’s disease found that CK treatment inhibited ATM/AMPK activation and reduced neuronal toxicity and mHTT aggregation. Most importantly, CK increased neuronal density and lifespan in R6/2 mice and improved motor dysfunction. Conversely, CK enhanced the expression of Bcl-2, which protects striatal cells from toxicity caused by mHTT and excessive AMPK activation (107).

### 5.4 Amyotrophic lateral sclerosis

Amyotrophic lateral sclerosis (ALS) is a progressive, fatal disease that affects both upper and lower motor neurons, leading to progressive muscle weakness, atrophy, and bulbar palsy, ultimately resulting in death from respiratory failure. For example, a study employed network pharmacology methods to systematically analyze the regulatory effects of ginseng catechins and ginsenosides on the aggregation of ALS-associated proteins. By constructing a catechin/ginsenoside-protein interaction network and comparing it with ALS transcriptomic datasets from the GEO database, the study found that knocking down catechin targets MAPK14, AKT, and GSK, as well as BACE1 in the ginsenoside-regulated pathway, effectively inhibits protein aggregation. The study indicates that catechins and ginsenosides have potential as therapeutic agents for ALS (108). Another *in vivo* study found that Gintonin prolonged survival in mice and alleviated motor dysfunction. Gintonin attenuated elevated expression of spinal cord NAD(P) quinone oxidoreductase 1 and reduced levels of oxidative stress-associated ferritin, ionized calcium-binding linker molecule 1-immunoreactive microglia, S100β-immunoreactive astrocytes, and Olig2-immunoreactive oligodendrocytes. Interestingly, the researchers found that gintonin treatment restored reduced LPA1 receptor expression levels, thereby alleviating neurological symptoms and histological defects (109). An *in vitro* study found that G-Rg1 reduced the apoptosis rate in SOD1G93A-NSC34 cells to some extent. Proteins associated with the mitochondrial apoptosis pathway, such as Bax, caspase 9 (Cas-9), and cytochrome c (Cyt c), were all downregulated. G-Rg1 also inhibits the activation of the nuclear factor-κB (NF-κB) signaling pathway by reducing the phosphorylation of p65 and IκBα (110). Li et al. found that ginsenoside compound K (CK) significantly inhibited the gelation of the GGGGCC repeat sequence in the ALS/frontotemporal dementia (FTD) gene C9orf72. It also reduced the co-aggregation of RNA with arginine-rich dipeptides through electrostatic interactions (111).

Overall, studies of the four NDD models indicate that the protective effects of ginseng’s active components are most consistent and reproducible in the AD and PD models, while the evidence is relatively weak in HD and ALS (Supplementary Table 1).

## 6 New strategies for delivering active components of ginseng

**Figure 5:**
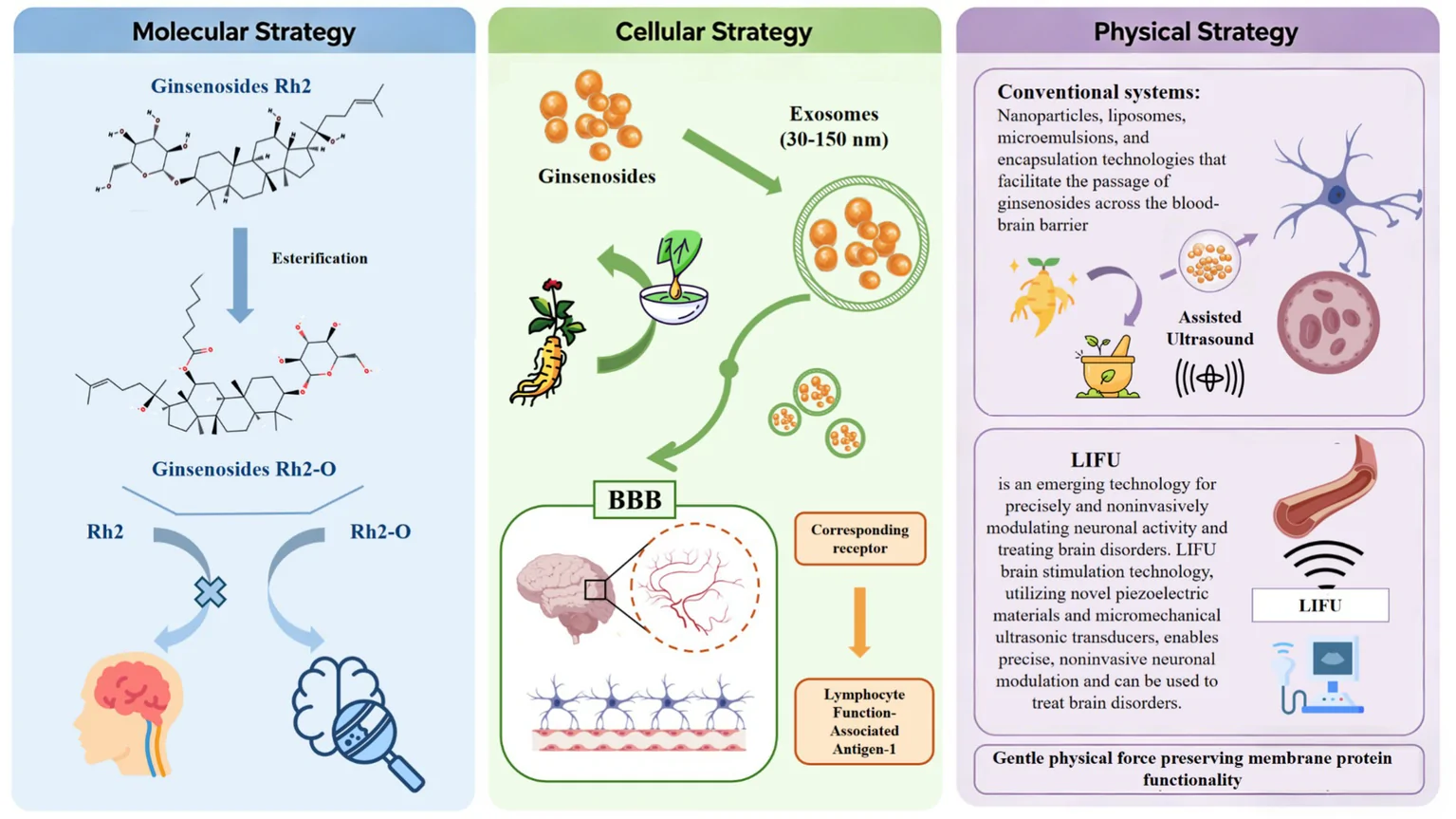
Three delivery strategies for ginseng-based targeted therapy of NDDs: Molecular, cellular, and physical. Image created with BioRender.com.

Ginseng and its bioactive components (particularly ginsenosides) have demonstrated therapeutic potential for various neurological disorders, including AD and PD, in numerous preclinical studies. However, a significant hurdle exists in translating the efficacy of these bioactive components into clinical applications, and at the core of this challenge lies the BBB. The BBB is a sophisticated system formed by brain capillary endothelial cells through tight junctions, supplemented by astrocyte dendrites, pericytes, and the basement membrane, which together form a barrier. It strictly regulates the entry of substances into the brain through physical, transport, and metabolic barriers, significantly limiting the effective delivery of highly water-soluble, high-molecular-weight ginseng bioactive components to the central nervous system, resulting in extremely low bioavailability in the brain (Figure 5). Therefore, developing strategies to efficiently and safely deliver ginseng’s active ingredients to brain tissue is the core challenge in realizing its neurotherapeutic value.

### 6.1 Molecular strategies

The core of molecular strategies lies in making drug molecules more capable of crossing the BBB by modifying their structure or leveraging their natural metabolic processes (112). Existing research has demonstrated that structural modifications not only enhance the biological activities of ginsenosides—such as their anticancer and anti-inflammatory effects—but also improve their absorption. In NDDs, impaired BBB function and oxidative damage to cerebral microvascular endothelial cells are critical factors contributing to neuronal death. Zhang et al. synthesized the prodrug Rh2-O by esterifying ginsenoside Rh2 (a PPD-type secondary saponin). This modification not only significantly improved the *in vitro* absorption of the ingredient but also demonstrated its potent protective effects against oxidative stress in Caco-2 and human umbilical vein endothelial cell models. This suggests that the prodrug Rh2-O may not only possess higher bioavailability than Rh2, but also exert protective effects on the vascular endothelium, implying its potential that it has the potential to directly stabilize and repair damaged BBB structures while crossing the BBB, thereby creating a more protective microenvironment for neurons and indirectly exerting neuroprotective effects (113). For example, the prodrug Rh2-O was synthesized by esterifying ginsenoside Rh2 (a PPD-type secondary ginsenoside). This modification not only significantly improved the ingredient’s in vitro absorption but also demonstrated its potent protective effects against oxidative stress in both Caco-2 and human umbilical vein endothelial cell models. This suggests that the prodrug Rh2-O may not only possess higher bioavailability than Rh2, but its protective effects on the vascular endothelium also imply that it has the potential to directly stabilize and repair damaged BBB structures while crossing the BBB, thereby creating a more protective microenvironment for neurons and indirectly exerting neuroprotective effects.

Following oral administration of ginsenosides, the actual substances circulating in the systemic circulation and the active forms are often secondary ginsenosides produced by the gut microbiota. These metabolites not only exhibit greater bioactivity but also typically possess physicochemical properties that facilitate their entry into the brain. Tang et al. screened edible fungal strains capable of converting less efficient glycosylated ginsenosides (Rb1, Rc, and Rb2) into highly efficient deglycosylated forms (Rd). They evaluated the anti-fatigue capacity and corresponding mechanisms of fermented ginsenoside extracts using an exercise-induced model, laying a solid foundation for the treatment of NDDs (114). Furthermore, we have found that many of the truly bioactive components of orally administered ginsenosides are metabolites produced by the gut microbiota. CK is the primary intestinal metabolite of the PPD type. Prebiotic intervention has demonstrated that it specifically enriches Prevotella species in the gut microbiota, thereby efficiently converting ginsenoside Rb1 into Compound K. Compared to Rb1, CK not only exhibits stronger neuroprotective activity, but its smaller molecular weight and higher lipophilicity also enable it to cross the blood–brain barrier more effectively, thereby acting directly on the central nervous system. This implies that by targeting the gut microbiota, we can transform oral Rb1 into a highly effective prodrug strategy, significantly enhancing its potential for combating NDDs (115). This suggested that developing CK or its efficient delivery system directly is an effective way to overcome the challenges associated with delivering protosaponins.

### 6.2 Cellular strategies

Cell-based strategies refer to approaches that utilize nanocarriers to mimic or hijack the body’s natural substance transport mechanisms to achieve BBB crossing. Drug delivery systems will significantly improve the bioavailability of ginsenosides while also enabling targeted, delayed, and controlled release. A 2025 study designed a ginsenoside Rg3-based vesicle (GRg3@RHT) for delivering rivastigmine hydrogen tartrate (RHT), a first-line clinical treatment for AD, resulting in a multi-targeted therapeutic effect (116). Phospholipid complexes represent a novel approach to vesicular drug delivery technology, utilizing phytochemicals and phospholipids to form micellar structures similar to liposomes. From a nanostructural perspective, the phytochemicals involved in plant systems can be considered substitutes for cholesterol—a regulator of membrane stability and fluidity in liposomal systems. The nanoscale size (approximately 100–200 nm) of these lipid-based nanoparticles facilitates transport to the olfactory and trigeminal nerves (117, 118). However, the blood–brain barrier (BBB) prevents most RHT from reaching the brain. Therefore, it is necessary to develop a nasal-to-brain drug delivery system using GRg3@RHT nasal spray (GRg3@RHT NS). Spherical gold nanoparticles (AuNPs) have been widely reported to exhibit biocompatibility in biological applications, particularly as drug carriers. In previous studies, AuNPs have been successfully synthesized as nanocarriers to deliver ginsenoside CK, thereby enhancing its water solubility and anticancer activity (119). Among nanomaterials used for encapsulation, chitosan (CS) is highly recommended as a carrier system due to its high loading capacity, stability, biodegradability, biocompatibility, and low immunogenicity (120). The synergistic effects of chitosan-ginsenoside CK-gold nanoparticles (CS-CK-AuNPs) result in enhanced anti-inflammatory activity. CS-CK-AuNPs significantly reduced the mRNA and protein expression of pro-inflammatory cytokines in LPS-induced RAW 264.7 cells. Additionally, CS-CK-AuNPs effectively inhibited the activation of the NF-κB signaling pathway (121).

Exosomes are naturally occurring nanovesicles secreted by cells that enter target cells via endocytosis or membrane fusion through interactions between their membrane proteins and the target cells. This represents a biomimetic, highly complex cellular strategy. In cell therapy strategies for NDDs, the ability of bone marrow mesenchymal stem cells (BMSCs) to differentiate into neural cells has been extensively explored. Ginseng-derived exosomes (G-Exos), as natural nanocarriers, can significantly promote the neural differentiation of BMSCs by delivering plant-derived miRNAs into them. In animal models, co-loading G-Exos with chemokines onto a photo-crosslinked hydrogel effectively recruits BMSCs to target areas and guides their neural differentiation, demonstrating application potential for cross-species regulation of stem cell differentiation in neural regenerative medicine (122). Xiaoxuming Decoction (XXMD-C) has demonstrated potential as an anti-neuroinflammatory agent. XXMD-C contains active components such as ginsenoside Rg1 and exerts anti-inflammatory effects in an LPS-induced inflammatory model of BV-2 microglia. Further studies revealed that XXMD-C exerts neuroprotective effects by regulating the expression of miR-9-5p in exosomes, thereby inhibiting key inflammatory factors such as IL-1β, IL-6, iNOS, and TNF-*α* (123).

### 6.3 Physical strategies

Ginsenosides are the primary active components of ginseng, a traditional Chinese medicine. They exhibit multi-targeted therapeutic effects and are associated with few side effects. Numerous preclinical studies have demonstrated their therapeutic value in conditions involving neurodiversity. Although oral absorption rates are low and permeability across the blood–brain barrier (BBB) is limited, research is currently underway on delivery systems such as nanoparticles, liposomes, and microemulsions to overcome these limitations (97).

Imtiaz et al. utilized focused ultrasound (FUS)-mediated blood–brain barrier (BBB) opening to enhance the delivery of Rg3-loaded nanoparticles in a mouse PD model induced by rohenone. Localized FUS ultrasound induced transient BBB disruption, followed by intraperitoneal administration of FITC-labeled Rg3 nanoparticles. *In vivo* and *in vitro* fluorescence imaging confirmed that nanoparticle accumulation in the brain significantly increased following FUS-mediated BBB opening. Treatment with Rg3 nanoparticles alone resulted in a moderate increase in both ATP levels and Complex I activity, while FUS alone showed a similar trend with a slightly higher recovery rate. Combination therapy with FUS-mediated BBB opening yielded higher mean values (ATP: 0.27 ± 0.06; Complex I: 1.98 ± 0.41) and improved motor performance compared to the PD group (124). Low-intensity focused ultrasound (LIFU) is an emerging technology for precisely and noninvasively modulating neuronal activity and treating brain disorders. LIFU brain stimulation technology, utilizing novel piezoelectric materials and micromechanical ultrasonic transducers, enables precise, noninvasive neuronal modulation and can be used to treat brain disorders such as AD and PD. The experiment employed a closed-loop system integrating ultrasound emission with real-time EEG monitoring, successfully modulating memory and motor function and clearing amyloid plaques in mouse and macaque models (125). Recent advancements in ultrasound brain stimulation technology have improved integration with existing biosensing systems, thereby facilitating a series of exploratory studies and bringing low-intensity focused ultrasound closer to clinical practice (126).

Nitrogen cavitation is a physical method of cell disruption. It utilizes the cavitation effect generated by the physical dissolution of gas and rapid pressure release as a physical force that is efficient, gentle, and capable of preserving the natural function of membrane proteins, thereby producing bioactive Neutrophil-like Cell Membrane Vesicles (NCM). The successful implementation of this physical strategy is the foundation and prerequisite for the entire biomimetic delivery system to achieve subsequent biological functions (such as inflammation targeting, penetration of the blood–brain barrier, and modulation of microglia). Ginsenoside Rg1 possesses neuroprotective properties and can regulate cellular metabolism *in vitro*; however, its therapeutic application is limited by poor brain penetration. Using NCMs enhances the delivery of Rg1 to the brain, where it reaches the site of brain injury and is subsequently taken up by microglia. Rg1-loaded vesicles enhance the microglia’s ability to clear neutrophils, reduce the release of extracellular neutrophil-derived debris, and mitigate brain tissue damage. Neuroinflammation is a key component of the brain cell death cascade. Activated microglia in the brain produce large amounts of inflammatory mediators and release pro-inflammatory cytokines, leading to disruption of the blood–brain barrier (BBB) and neuronal damage. NCM exhibits strong inflammation-targeting properties and effectively aids Rg1 in crossing the BBB, thereby increasing Rg1 levels in the brain (127).

Among the three delivery strategies, extracellular vesicles—due to their natural targeting ability, low immunogenicity, and dual function of carrying endogenous active miRNAs—have demonstrated comprehensive advantages over synthetic nanoparticles and traditional physical methods in crossing the blood–brain barrier and regulating neuroinflammation. Although physical strategies show promise, they are highly equipment-dependent and exhibit significant interindividual variability in their activation and deactivation effects, and thus remain some distance from routine clinical application. It should be noted that the *in vivo* evaluation of the aforementioned novel delivery strategy was conducted in mouse or rat models. There has been no systematic assessment of whether its ability to open the blood–brain barrier is reproducible in the human brain or of its long-term safety (128, 129).

## 7 Conclusion

In summary, ginseng contains various bioactive components, including saponins, polysaccharides, essential oils, and peptides. It exerts its anti-NDD effects through mechanisms such as regulating protein homeostasis, alleviating neuroinflammation, inhibiting excitotoxicity, and improving mitochondrial oxidative stress. Specifically, it has demonstrated neuroprotective potential in models of NDDs such as AD, PD, HD, and ALS. Concurrently, the development of novel delivery strategies (including molecular, cellular, and physical approaches) offers viable pathways to overcome the bottlenecks associated with poor blood–brain barrier permeability and low bioavailability of ginseng’s active components. However, current research still faces significant limitations: most evidence is concentrated in cellular and animal models, and there is a severe lack of high-quality clinical studies. The mechanisms underlying the synergistic interactions among active components remain unclear. Furthermore, the long-term safety of novel delivery systems and their scalable manufacturing processes require validation. The vast majority of the findings summarized in this narrative review are still in the preclinical validation stage, with most studies focusing on animal models and in vitro experiments. Future research should focus on conducting clinical trials, utilizing systems biology and multi-omics technologies to elucidate the regulatory mechanisms of ginseng, and promoting the clinical translation and personalized application of targeted delivery systems. This will lay a solid foundation for the practical application of ginseng in the prevention and treatment of NDDs.

## References

[1] Wilson DM 3rd Cookson MR Van Den Bosch L Zetterberg H Holtzman DM Dewachter I. Hallmarks of neurodegenerative diseases. Cell. (2023) 186:693–714. doi: 10.1016/j.cell.2022.12.032, 36803602

[2] Liu Y Hu Z Xiao Y Han X Cheng X Ye T. Microglial senescence in neurodegenerative diseases: mechanisms and therapeutic perspectives. ACS Chem Neurosci. (2026) 17:1088–103. doi: 10.1021/acschemneuro.6c00016, 41735187

[3] Shomali T Trempe JF. Targeting of kinases to treat neurodegenerative diseases. Pharmacol Rev. (2026) 78:100128. doi: 10.1016/j.pharmr.2026.100128, 41880688 PMC13197926

[4] Goyal R Mittal P Gautam RK Kamal MA Perveen A Garg V . Natural products in the management of neurodegenerative diseases. Nutr Metab. (2024) 21:26. doi: 10.1186/s12986-024-00800-4, 38755627 PMC11100221

[5] Ye X-S Tian W-J Wang G-H Lin K Zhu S-X Xia Y-Y . The food and medicine homologous Chinese medicine from Leguminosae species: a comprehensive review on bioactive constituents with neuroprotective effects on nervous system. Food Med Homol. (2025) 2:9420033. doi: 10.26599/fmh.2025.9420033

[6] Zhang Y-M Wang G. A comparative study of goji berry and ashwagandha extracts effects on promoting neural stem cell proliferation and reducing neuronal cell death. Food Med Homol. (2025) 2:9420039. doi: 10.26599/fmh.2025.9420039

[7] Mehrnoosh F Rezaei D Pakmehr SA Nataj PG Sattar M Shadi M . The role of Panax ginseng in neurodegenerative disorders: mechanisms, benefits, and future directions. Metab Brain Dis. (2025) 40:183. doi: 10.1007/s11011-025-01610-0, 40232582

[8] Yue J Zuo Z Huang H Wang Y. Application of identification and evaluation techniques for ethnobotanical medicinal Plant of Genus Panax: a review. Crit Rev Anal Chem. (2021) 51:373–98. doi: 10.1080/10408347.2020.1736506, 32166968

[9] Guo J Zhao X-Y Cao Y-X Jia J-H Zheng Y-F Sun W-L . The fundamental traditional Chinese medicine constitution theory serves as a crucial basis for the development and application of food and medicine homology products. Food Med Homol. (2025) 2:9420122. doi: 10.26599/fmh.2025.9420122

[10] Zhang Z-B Yu C-Y Wang H-Y Jia X-B Wu W Pang S-T . The history, beneficial ingredients, mechanism, processing, and products of *Panax ginseng* for medicinal and edible value. Food Med Homol. (2025) 2:9420059. doi: 10.26599/fmh.2025.9420059

[11] Attele AS Wu JA Yuan CS. Ginseng pharmacology: multiple constituents and multiple actions. Biochem Pharmacol. (1999) 58:1685–93. doi: 10.1016/S0006-2952(99)00212-9, 10571242

[12] Gadhave DG Sugandhi VV Jha SK Nangare SN Gupta G Singh SK . Neurodegenerative disorders: mechanisms of degeneration and therapeutic approaches with their clinical relevance. Ageing Res Rev. (2024) 99:102357. doi: 10.1016/j.arr.2024.102357, 38830548

[13] Zhai K Duan H Wang W Zhao S Khan GJ Wang M . Ginsenoside Rg1 ameliorates blood-brain barrier disruption and traumatic brain injury via attenuating macrophages derived exosomes miR-21 release. Acta Pharm Sin B. (2021) 11:3493–507. doi: 10.1016/j.apsb.2021.03.032, 34900532 PMC8642604

[14] Sun M Li Y Zhu M Luo H Teng Y. Ginsenosides in modern pharmaceutics: mechanisms, applications, challenges, and perspectives. Biomolecules. (2026) 16:350. doi: 10.3390/biom16030350, 41897289 PMC13024219

[15] Yu W Cai S Zhao J Hu S Zang C Xu J . Beyond genome: advanced omics progress of *Panax ginseng*. Plant Sci. (2024) 341:112022. doi: 10.1016/j.plantsci.2024.112022, 38311250

[16] Wang CZ Zhang CF Zhang QH Yuan CS. Phytochemistry of red ginseng, a steam-processed *Panax ginseng*. Am J Chin Med. (2024) 52:35–55. doi: 10.1142/S0192415X24500022, 38353635

[17] Huang Q Gao S Zhao D Li X. Review of ginsenosides targeting mitochondrial function to treat multiple disorders: current status and perspectives. J Ginseng Res. (2021) 45:371–9. doi: 10.1016/j.jgr.2020.12.004, 34025130 PMC8134842

[18] Gong PX Zong WL Li HH Wu YC Ju H Fan ZW . Comprehensive analysis of different types of ginsenosides in the different parts of American ginseng by targeted and nontargeted MS/MS scanning. J Food Sci. (2023) 88:5063–77. doi: 10.1111/1750-3841.16821, 37921543

[19] You L Cha S Kim MY Cho JY. Ginsenosides are active ingredients in *Panax ginseng* with immunomodulatory properties from cellular to organismal levels. J Ginseng Res. (2022) 46:711–21. doi: 10.1016/j.jgr.2021.12.007, 36312737 PMC9597430

[20] Sun XB Gao DY Cao JW Liu Y Rong ZT Wang JK . Bslpmo10A from *Bacillus subtilis* boosts the depolymerization of diverse polysaccharides linked via β-1,4-glycosidic bonds. Int J Biol Macromol. (2023) 230:123133. doi: 10.1016/j.ijbiomac.2023.123133, 36621733

[21] Hu Y He Y Niu Z Shen T Zhang J Wang X . A review of the immunomodulatory activities of polysaccharides isolated from Panax species. J Ginseng Res. (2022) 46:23–32. doi: 10.1016/j.jgr.2021.06.003, 35058724 PMC8753523

[22] Wang N Wang X He M Zheng W Qi D Zhang Y . Ginseng polysaccharides: a potential neuroprotective agent. J Ginseng Res. (2021) 45:211–7. doi: 10.1016/j.jgr.2020.09.002, 33841001 PMC8020291

[23] Xu Y Bian S Shang L Wang X Bai X Zhang W. Phytochemistry, pharmacological effects and mechanism of action of volatile oil from *Panax ginseng* C.A.Mey: a review. Front Pharmacol. (2024) 15:1436624. doi: 10.3389/fphar.2024.1436624, 39193331 PMC11347760

[24] Yoon SJ Sukweenadhi J Khorolragchaa A Mathiyalagan R Subramaniyam S Kim YJ . Overexpression of *Panax ginseng* sesquiterpene synthase gene confers tolerance against *Pseudomonas syringae* pv. Tomato in *Arabidopsis thaliana*. Physiol Mol Biol Plants. (2016) 22:485–95. doi: 10.1007/s12298-016-0384-9, 27924121 PMC5120041

[25] Sun H Liu F Sun L Liu J Wang M Chen X . Proteomic analysis of amino acid metabolism differences between wild and cultivated *Panax ginseng*. J Ginseng Res. (2016) 40:113–20. doi: 10.1016/j.jgr.2015.06.001, 27158231 PMC4845045

[26] Liu Z Wen X Wang CZ Li W Huang WH Xia J . Remarkable impact of amino acids on ginsenoside transformation from fresh ginseng to red ginseng. J Ginseng Res. (2020) 44:424–34. doi: 10.1016/j.jgr.2019.04.001, 32372864 PMC7195590

[27] Gstirner F Vogt HJ. On peptides in white Korean ginseng. Arch Pharm Ber Dtsch Pharm Ges. (1966) 299:936–44. doi: 10.1002/ardp.19662991108, 5232068

[28] Liu T Zhu C Duan Z Ma P Ma X Fan D. Network pharmacological analysis combined with experimental verification to explore the effect of ginseng polypeptide on the improvement of diabetes symptoms in db/db mice. J Agric Food Chem. (2024) 72:18537–51. doi: 10.1021/acs.jafc.4c04949, 39129180

[29] Liu Z Liu X Li W Luo Q Liu J Wang D. Anti-colon cancer activity tracking isolation of peptide from ginseng leaves and potential mechanisms evaluation in vitro and *in vivo*. J Pept Sci. (2021) 27:e3297. doi: 10.1002/psc.3297, 33462944

[30] Zhao N Cheng M Wu Y Liu D Zhang X. Differential analysis of peptides in *Panax ginseng* C. A. Meyer root by ultra-performance liquid chromatography-high resolution mass spectrometry. Se Pu. (2019) 37:1305–13. doi: 10.3724/SP.J.1123.2019.09006, 34213132

[31] Bu K Cizdziel JV Reidy L. Analysis of herbal supplements for selected dietary minerals and trace elements by laser ablation- and solution-based ICPMS. Microchem J. (2013) 106:244–9. doi: 10.1016/j.microc.2012.07.011

[32] Yeo CR Yong JJ Popovich DG. Isolation and characterization of bioactive polyacetylenes *Panax ginseng* Meyer roots. J Pharm Biomed Anal. (2017) 139:148–55. doi: 10.1016/j.jpba.2017.02.054, 28282601

[33] Resetar M Liu X Herdlinger S Kunert O Pferschy-Wenzig EM Latkolik S . Polyacetylenes from Oplopanax horridus and *Panax ginseng*: relationship between structure and PPARγ activation. J Nat Prod. (2020) 83:918–26. doi: 10.1021/acs.jnatprod.9b00691, 32129622 PMC7187397

[34] Gupta R Sahu M Srivastava D Tiwari S Ambasta RK Kumar P. Post-translational modifications: regulators of neurodegenerative proteinopathies. Ageing Res Rev. (2021) 68:101336. doi: 10.1016/j.arr.2021.101336, 33775891

[35] Pandey LM. Physicochemical factors of bioprocessing impact the stability of therapeutic proteins. Biotechnol Adv. (2022) 55:107909. doi: 10.1016/j.biotechadv.2022.107909, 35031395

[36] Chen L Shen Q Liu Y Zhang Y Sun L Ma X . Homeostasis and metabolism of iron and other metal ions in neurodegenerative diseases. Signal Transduct Target Ther. (2025) 10:31. doi: 10.1038/s41392-024-02071-0, 39894843 PMC11788444

[37] Cho YY Park JH Lee JH Chung S. Ginsenosides decrease β-amyloid production via potentiating capacitative calcium entry. Biomol Ther. (2024) 32:301–8. doi: 10.4062/biomolther.2023.172, 38586949 PMC11063476

[38] Xu N Xing S Li J Pang B Liu M Fan M . Water extract of ginseng alleviates parkinsonism in MPTP–induced Parkinson’s disease mice. PLoS One. (2024) 19:e0296424. doi: 10.1371/journal.pone.0296424, 39302939 PMC11414931

[39] Li J Zhao G Yu C Qu Y Shen X Zhao Y . Ginsenoside re exerts neuroprotective in MPTP mice: potential links to gut microbiota and serum metabolism. Neuropharmacology. (2026) 282:110596. doi: 10.1016/j.neuropharm.2025.110596, 40721043

[40] Niu H Zhang M Zhang K Aishan S Li H Wu W. In-depth investigation on potential mechanism of forest-grown ginseng alleviating Alzheimer’s disease via UHPLC-MS-based metabolomics. Meta. (2025) 15:93. doi: 10.3390/metabo15020093, 39997718 PMC11857256

[41] Wang N Yang J Chen R Liu Y Liu S Pan Y . Ginsenoside Rg1 ameliorates Alzheimer's disease pathology via restoring mitophagy. J Ginseng Res. (2023) 47:448–57. doi: 10.1016/j.jgr.2022.12.001, 37252274 PMC10214288

[42] Shalaby AM Alnasser SM Ahmed Khairy D Alabiad MA Alorini M Jaber FA . The neuroprotective effect of ginsenoside Rb1 on the cerebral cortex changes induced by aluminium chloride in a mouse model of Alzheimer's disease: a histological, immunohistochemical, and biochemical study. J Chem Neuroanat. (2023) 129:102248. doi: 10.1016/j.jchemneu.2023.102248, 36764334

[43] Uddin MS Yu WS Lim LW. Exploring ER stress response in cellular aging and neuroinflammation in Alzheimer's disease. Ageing Res Rev. (2021) 70:101417. doi: 10.1016/j.arr.2021.101417, 34339860

[44] Wang H Liu S Sun Y Chen C Hu Z Li Q . Target modulation of glycolytic pathways as a new strategy for the treatment of neuroinflammatory diseases. Ageing Res Rev. (2024) 101:102472. doi: 10.1016/j.arr.2024.102472, 39233146

[45] Ferrer I. Diversity of astroglial responses across human neurodegenerative disorders and brain aging. Brain Pathol. (2017) 27:645–74. doi: 10.1111/bpa.12538, 28804999 PMC8029391

[46] Jing N Li X. Dihydromyricetin attenuates inflammation through TLR4/NF-kappaB pathway. Open Med. (2019) 14:719–25. doi: 10.1515/med-2019-0083, 31572805 PMC6749725

[47] La Vitola P Balducci C Cerovic M Santamaria G Brandi E Grandi F . Alpha-synuclein oligomers impair memory through glial cell activation and via toll-like receptor 2. Brain Behav Immun. (2018) 69:591–602. doi: 10.1016/j.bbi.2018.02.012, 29458199

[48] Mcmanus RM Latz E. NLRP3 inflammasome signalling in Alzheimer's disease. Neuropharmacology. (2024) 252:109941. doi: 10.1016/j.neuropharm.2024.109941, 38565393

[49] Pascual M Ibáñez F Guerri C. Exosomes as mediators of neuron-glia communication in neuroinflammation. Neural Regen Res. (2020) 15:796–801. doi: 10.4103/1673-5374.268893, 31719239 PMC6990780

[50] Chen Y Meng J Xu Q Long T Bi F Chang C . Rapamycin improves the neuroprotection effect of inhibition of NLRP3 inflammasome activation after TBI. Brain Res. (2019) 1710:163–72. doi: 10.1016/j.brainres.2019.01.005, 30615886

[51] Matsumoto J Dohgu S Takata F Machida T Bölükbaşi Hatip FF Hatip-Al-Khatib I . TNf-α-sensitive brain pericytes activate microglia by releasing Il-6 through cooperation between IκB-NFκB and JAK-STAT3 pathways. Brain Res. (2018) 1692:34–44. doi: 10.1016/j.brainres.2018.04.023, 29702085

[52] Soylu-Kucharz R Khoshnan A Petersén Å. IKKβ signaling mediates metabolic changes in the hypothalamus of a Huntington disease mouse model. iScience. (2022) 25:103771. doi: 10.1016/j.isci.2022.103771, 35146388 PMC8819015

[53] Li D Yang H Ma J Luo S Chen S Gu Q. Microrna-30e regulates neuroinflammation in MPTP model of Parkinson's disease by targeting Nlrp3. Hum Cell. (2018) 31:106–15. doi: 10.1007/s13577-017-0187-5, 29274035 PMC5852205

[54] Jiang M Chi J Qiao Y Wang J Zhang Z Liu J . Ginsenosides Rg1, Rb1 and rare ginsenosides: promising candidate agents for Parkinson's disease and Alzheimer's disease and network pharmacology analysis. Pharmacol Res. (2025) 212:107578. doi: 10.1016/j.phrs.2025.107578, 39756554

[55] Li T Chen J Xie Z Fang J Wu Q Cao X . Ginsenoside Ro ameliorates cognitive impairment and neuroinflammation in App/PS1 mice via the IBA1/GFAP-MAPK signaling pathway. Front Pharmacol. (2025) 16:1528590. doi: 10.3389/fphar.2025.1528590 40066331 PMC11891224

[56] Dilida Y Yue G Jing C Qianqian Z Gong W Yifa Z . Ginsenoside Rg2 ameliorates Alzheimer's disease by alleviating neuroinflammation in APP/PS1 mice. Curr Neuropharmacol. (2025) 23:1–13. doi: 10.2174/011570159x395496250911104139 41029023

[57] Madhi I Kim J-H Shin HS So YH Jung E-M Kim Y. Ginsenoside re mitigates Aβ1-42-induced neurotoxicity by promoting autophagy and suppressing Nlrp3 inflammasome. J Ginseng Res. (2026) 50:100911. doi: 10.1016/j.jgr.2025.10.008, 41550591 PMC12805552

[58] Wang Y Zhang Y Li Y Zhang Z Lian X-Y. The necessity of eliminating the interference of panaxatriol saponins to maximize the preventive effect of panaxadiol saponins against Parkinson's disease in rats. J Ginseng Res. (2024) 48:464–73. doi: 10.1016/j.jgr.2024.05.002, 39263312 PMC11385176

[59] Armada-Moreira A Gomes JI Pina CC Savchak OK Gonçalves-Ribeiro J Rei N . Going the extra (synaptic) mile: Excitotoxicity as the road toward neurodegenerative diseases. Front Cell Neurosci. (2020) 14:90. doi: 10.3389/fncel.2020.00090, 32390802 PMC7194075

[60] Doig AJ. Positive feedback loops in Alzheimer's disease: the Alzheimer's feedback hypothesis. J Alzheimer's Dis. (2018) 66:25–36. doi: 10.3233/JAD-180583, 30282364 PMC6484277

[61] Lu KW Yang J Hsieh CL Hsu YC Lin YW. Electroacupuncture restores spatial learning and downregulates phosphorylated N-methyl-D-aspartate receptors in a mouse model of Parkinson's disease. Acupunct Med. (2017) 35:133–41. doi: 10.1136/acupmed-2015-011041, 27531695

[62] Oskarsson B Mauricio EA Shah JS Li Z Rogawski MA. Cortical excitability threshold can be increased by the AMPA blocker Perampanel in amyotrophic lateral sclerosis. Muscle Nerve. (2021) 64:215–9. doi: 10.1002/mus.27328, 34008857

[63] Fan J Gladding CM Wang L Zhang LY Kaufman AM Milnerwood AJ . P38 MAPK is involved in enhanced NMDA receptor-dependent excitotoxicity in YAC transgenic mouse model of Huntington disease. Neurobiol Dis. (2012) 45:999–1009. doi: 10.1016/j.nbd.2011.12.019, 22198502

[64] Fan MM Fernandes HB Zhang LY Hayden MR Raymond LA. Altered NMDA receptor trafficking in a yeast artificial chromosome transgenic mouse model of Huntington's disease. J Neurosci. (2007) 27:3768–79. doi: 10.1523/JNEUROSCI.4356-06.2007, 17409241 PMC6672397

[65] Zhang H Li Q Graham RK Slow E Hayden MR Bezprozvanny I. Full length mutant huntingtin is required for altered Ca2+ signaling and apoptosis of striatal neurons in the YAC mouse model of Huntington's disease. Neurobiol Dis. (2008) 31:80–8. doi: 10.1016/j.nbd.2008.03.010, 18502655 PMC2528878

[66] Zhang M Niu H Li Q Jiao L Li H Wu W. Active compounds of *Panax ginseng* in the improvement of Alzheimer’s disease and application of spatial metabolomics. Pharmaceuticals. (2024) 17:38. doi: 10.3390/ph17010038, 38256872 PMC10818864

[67] Zieneldien T Kim J Cao C. The multifaceted role of neuroprotective plants in Alzheimer's disease treatment. Geriatrics. (2022) 7:24. doi: 10.3390/geriatrics7020024, 35314596 PMC8938774

[68] Li Q Rubin L Silva M Li S Yang C Lazarovici P . Current Progress on neuroprotection induced by Artemisia, ginseng, Astragalus, and Ginkgo traditional Chinese medicines for the therapy of Alzheimer's disease. Oxidative Med Cell Longev. (2022) 2022:3777021. doi: 10.1155/2022/3777021, 35746960 PMC9213169

[69] Nguyen YND Jeong JH Sharma N Tran NKC Tran H-YP Dang D-K . Ginsenoside re protects against kainate-induced neurotoxicity in mice by attenuating mitochondrial dysfunction through activation of the signal transducers and activators of transcription 3 signaling. Free Radic Res. (2024) 58:276–92. doi: 10.1080/10715762.2024.2341885, 38613520

[70] Tran NKC Jeong JH Sharma N Nguyen YND Tran H-YP Dang D-K . Ginsenoside re blocks Bay k-8644-induced neurotoxicity via attenuating mitochondrial dysfunction and PKCδ activation in the hippocampus of mice: involvement of antioxidant potential. Food Chem Toxicol. (2023) 178:113869. doi: 10.1016/j.fct.2023.113869, 37308051

[71] Ashleigh T Swerdlow RH Beal MF. The role of mitochondrial dysfunction in Alzheimer's disease pathogenesis. Alzheimers Dement. (2023) 19:333–42. doi: 10.1002/alz.12683, 35522844

[72] Yu Y-B Zhang Q Guo Y-H Wu J-J Zhao L-X Wang Z . ROS-related nanoparticles for the management of Alzheimer’s disease: wielding the double-edged sword. Chem Eng J. (2025) 511:161784. doi: 10.1016/j.cej.2025.161784

[73] Fišar Z Hansíková H KříŽová J Jirák R Kitzlerová E Zvěřová M . Activities of mitochondrial respiratory chain complexes in platelets of patients with Alzheimer's disease and depressive disorder. Mitochondrion. (2019) 48:67–77. doi: 10.1016/j.mito.2019.07.013, 31377247

[74] Csaban D Pentelenyi K Toth-Bencsik R Illes A Grosz Z Gezsi A . The role of the rare variants in the genes encoding the alpha-ketoglutarate dehydrogenase in Alzheimer's disease. Life. (2021) 11:321. doi: 10.3390/life11040321, 33917565 PMC8067443

[75] Chakraborty A Bose R Bose K. Unraveling the dichotomy of enigmatic serine protease HtrA2. Front Mol Biosci. (2022) 9:824846. doi: 10.3389/fmolb.2022.824846, 35187085 PMC8850690

[76] Mani S Sevanan M Krishnamoorthy A Sekar S. A systematic review of molecular approaches that link mitochondrial dysfunction and neuroinflammation in Parkinson's disease. Neurol Sci. (2021) 42:4459–69. doi: 10.1007/s10072-021-05551-1, 34480241

[77] Eisermann J Liang Y Wright JJ Clifford E Wilton-Ely J Kuimova MK . The effect of reactive oxygen species on respiratory complex I activity in liposomes. Chemistry. (2024) 30:e202402035. doi: 10.1002/chem.202402035, 39058376

[78] Elfawy HA Das B. Crosstalk between mitochondrial dysfunction, oxidative stress, and age related neurodegenerative disease: etiologies and therapeutic strategies. Life Sci. (2019) 218:165–84. doi: 10.1016/j.lfs.2018.12.029, 30578866

[79] Perera ND Sheean RK Lau CL Shin YS Beart PM Horne MK . Rilmenidine promotes MTOR-independent autophagy in the mutant SOD1 mouse model of amyotrophic lateral sclerosis without slowing disease progression. Autophagy. (2018) 14:534–51. doi: 10.1080/15548627.2017.1385674, 28980850 PMC5915012

[80] Lopes C Ferreira IL Maranga C Beatriz M Mota SI Sereno J . Mitochondrial and redox modifications in early stages of Huntington's disease. Redox Biol. (2022) 56:102424. doi: 10.1016/j.redox.2022.102424, 35988447 PMC9420526

[81] Okada N Yako T Nakamura S Shimazawa M Hara H. Reduced mitochondrial complex ii activity enhances cell death via intracellular reactive oxygen species in SthdhQ111 striatal neurons with mutant huntingtin. J Pharmacol Sci. (2021) 147:367–75. doi: 10.1016/j.jphs.2021.09.001, 34663519

[82] Li Z Wang C Wang Z Zhu C Li J Sha T . Allele-selective lowering of mutant HTT protein by HTT-LC3 linker compounds. Nature. (2019) 575:203–9. doi: 10.1038/s41586-019-1722-1, 31666698

[83] Liu Z Cecarini V Cuccioloni M Bonfili L Gong C Angeletti M . Ginsenosides Rg1 and Rg2 activate autophagy and attenuate oxidative stress in neuroblastoma cells overexpressing Aβ(1-42). Antioxidants. (2024) 13:310. doi: 10.3390/antiox13030310, 38539843 PMC10967604

[84] Quan Q Ma X Feng J Li W Li X. Ginsenoside Rg1 improves autophagy dysfunction to ameliorate Alzheimer's disease via targeting FGR proto-oncogene. Neuropeptides. (2025) 111:102514. doi: 10.1016/j.npep.2025.102514, 40073763

[85] Zhao J Wang J Zhao K Zhang Y Hu W. Protopanaxadiols eliminate behavioral impairments and mitochondrial dysfunction in Parkinson’s disease mice model. Neurochem Res. (2024) 49:1751–61. doi: 10.1007/s11064-024-04132-w, 38551796 PMC11144128

[86] Zhao C Yue J Xie Y Liu B Xu S Zhi D . A Ginsenoside composition ameliorated aβ and tau aggregation via autophagy lysosome pathway. Mol Neurobiol. (2025) 62:11822–33. doi: 10.1007/s12035-025-05017-x, 40327308

[87] Zhao X Yin H Du R He Z Pei H. Glycation aging environment: abnormal glycosylation and advanced glycation end products drive neural aging. Neural Regen Res. (2026). doi: 10.4103/nrr.nrr-d-25-01586 42199115

[88] Barani M Zargari F Mirinejad S Madani F Hajinezhad MR Sargazi S. Ginsenoside Rg3-encapsulated pegylated niosomes exhibit multimodal therapeutic potential in Alzheimer’s disease. Sci Rep. (2025) 16:2547. doi: 10.1038/s41598-025-32528-3, 41392105 PMC12820278

[89] Xie C Zhang C Zhang K Zhang S. Ginsenoside C-K inhibits Aβ oligomer-induced Alzheimer's disease pathology progression by regulating microglia-neuron interactions. Ibro Neurosci Rep. (2025) 18:783–93. doi: 10.1016/j.ibneur.2025.05.007, 40510292 PMC12159499

[90] Zhang Y Liu S Cao D Zhao M Lu H Wang P. Rg1 improves Alzheimer's disease by regulating mitochondrial dynamics mediated by the AMPK/Drp1 signaling pathway. J Ethnopharmacol. (2025) 340:119285. doi: 10.1016/j.jep.2024.119285, 39733799

[91] Fang H Tian H Liu J Peng T Wang D. Ginsenoside Rg1 attenuates Aβ1-42-induced microglial cell apoptosis and inflammation in Alzheimer’s disease via the GATA4/PDE4A/PI3K/AKT axis. Neuroscience. (2025) 565:377–85. doi: 10.1016/j.neuroscience.2024.12.011, 39653247

[92] Lu Y-W Wang Y-J Wang Z Ren S Gong X-J Hu J-N . Ginsenoside Rg2 alleviates astrocyte inflammation and ameliorates the permeability of the Alzheimer's disease related blood-brain barrier. Phytomedicine. (2024) 135:156063. doi: 10.1016/j.phymed.2024.156063, 39305744

[93] Yin P Wang H Xue T Yu X Meng X Mi Q . Four-dimensional label-free quantitative proteomics of Ginsenoside Rg2 ameliorated scopolamine-induced memory impairment in mice through the lysosomal pathway. J Agric Food Chem. (2024) 72:14640–52. doi: 10.1021/acs.jafc.4c00181, 38885433

[94] He S Shi J Ma L Pei H Zhang P Shi D . Total ginsenosides decrease Aβ production through activating PPARγ. Biomed Pharmacother. (2024) 174:116577. doi: 10.1016/j.biopha.2024.116577, 38593704

[95] Quan Q Ma X Li M Li X Yuan H. Ginsenoside Rg1 promotes β-amyloid peptide degradation through inhibition of the ERK/PPARγ phosphorylation pathway in an Alzheimer's disease neuronal model. Exp Ther Med. (2024) 27:31. doi: 10.3892/etm.2023.12319, 38125359 PMC10731411

[96] She L Tang H Zeng Y Li L Xiong L Sun J . Ginsenoside RK3 promotes neurogenesis in Alzheimer's disease through activation of the CREB/BDNF pathway. J Ethnopharmacol. (2024) 321:117462. doi: 10.1016/j.jep.2023.117462, 37981117

[97] Ding Y Botchway BOA Zhang Y Liu X. Ginsenosides can target brain-derived neurotrophic factor to improve Parkinson's disease. Food Funct. (2023) 14:5537–50. doi: 10.1039/D2FO03484K, 37279045

[98] Sun Y Xu N Liu J Sun T Li J Xing S . Protective effect of ginseng protein on MPTP-induced Parkinson’s disease model mice. Nat Prod Res. (2026):1–11. doi: 10.1080/14786419.2026.2636160 41749513

[99] Yang X Zhao Y Liang L Qu Y Yu C Zhang J . Protective effect of ginsenoside CK against MPTP-induced Parkinson’ s disease mouse model by suppressing oxidative stress and inflammation, and modulating the gut microbiota. Microb Pathog. (2025) 202:107409. doi: 10.1016/j.micpath.2025.107409, 40010656

[100] Zhou S Liu Y Liu Y Duan Z Zhu C Wang P . Ginsenoside Rk3 alleviates Neuroinflammation and gastrointestinal dysfunction in Parkinson’s disease via modulation of gut microbiota-mediated butyric acid metabolism. J Agric Food Chem. (2025) 73:24139–54. doi: 10.1021/acs.jafc.5c06844, 40947628

[101] Wang Q Wei L Chen G Chen Q. Ginsenoside Rg1 in Parkinson’s disease: from basic research to clinical applications. Front Pharmacol. (2025) 16:1490480. doi: 10.3389/fphar.2025.1490480 40308780 PMC12040930

[102] Wang S-S Peng Y Fan P-L Ye J-R Ma W-Y Wu Q-L . Ginsenoside Rg1 ameliorates stress-exacerbated Parkinson’s disease in mice by eliminating RTP801 and α-synuclein autophagic degradation obstacle. Acta Pharmacol Sin. (2025) 46:308–25. doi: 10.1038/s41401-024-01374-w, 39227736 PMC11747340

[103] Jin C Qiao J Gong H Yu X Wang X Xi J . Ginsenoside re binds Drp1 Leu94 across species to restore mitochondrial homeostasis via Atg1/ULK1-dependent fission-mitophagy coupling in age-related degeneration. Pharmacol Res. (2025) 222:108043. doi: 10.1016/j.phrs.2025.108043, 41275989

[104] Qi L-F-R Liu S Fang Q Qian C Peng C Liu Y . Ginsenoside Rg3 restores mitochondrial Cardiolipin homeostasis via GRB2 to prevent Parkinson's disease. Adv Sci. (2024) 11:e2403058. doi: 10.1002/advs.202403058, 39159293 PMC11497058

[105] Lee M Ban J-J Won BH Im W Kim M. Therapeutic potential of ginsenoside Rg3 and Rf for Huntington’s disease. In Vitro Cell Dev Biol - Anim. (2021) 57:641–8. doi: 10.1007/s11626-021-00595-1, 34128157

[106] Subin JA Shrestha RLS. Computational assessment of the phytochemicals of *Panax ginseng* C.A. Meyer against dopamine receptor D1 for early Huntington's disease prophylactics. Cell Biochem Biophys. (2024) 82:3413–23. doi: 10.1007/s12013-024-01426-2, 39046621

[107] Hua K-F Chao AC Lin T-Y Chen W-T Lee Y-C Hsu W-H . Ginsenoside compound K reduces the progression of Huntington's disease via the inhibition of oxidative stress and overactivation of the ATM/AMPK pathway. J Ginseng Res. (2022) 46:572–84. doi: 10.1016/j.jgr.2021.11.003, 35818427 PMC9270658

[108] Swaroop RS Pradhan SS Darshan VMD Phalguna KS Sivaramakrishnan V. Integrated network pharmacology approach shows a potential role of ginseng catechins and ginsenosides in modulating protein aggregation in amyotrophic lateral sclerosis. 3 Biotech. (2022) 12:333. doi: 10.1007/s13205-022-03401-1, 36330377 PMC9622974

[109] Nam SM Choi JH Choi S-H Cho H-J Cho Y-J Rhim H . Ginseng gintonin alleviates neurological symptoms in the G93A-Sod1 transgenic mouse model of amyotrophic lateral sclerosis through lysophosphatidic acid 1 receptor. J Ginseng Res. (2021) 45:390–400. doi: 10.1016/j.jgr.2020.04.002, 34025132 PMC8134849

[110] Huang Y Yang H Yang B Zheng Y Hou X Chen G . Ginsenoside-Rg1 combined with a conditioned medium from induced neuron-like hUCMSCs alleviated the apoptosis in a cell model of ALS through regulating the NF-κB/Bcl-2 pathway. Chin J Nat Med. (2023) 21:540–50. doi: 10.1016/S1875-5364(23)60445-5, 37517821

[111] Li Y Liu S Cao L Zhu M Lin S Wang X . Ginsenoside compound K inhibited the gelation of GGGGCC repeats and regulated co-aggregation with arginine-rich poly-dipeptides in C9orf72-related Als. Int J Biol Macromol. (2026) 351:151138. doi: 10.1016/j.ijbiomac.2026.151138, 41763422

[112] Zhu B-R. Spatial metabolomics: revolutionary transformation in food and medicinal homology research—decoding the spatiotemporal map of food and medicinal substances in organisms. Food Med Homol. (2025) 2:9420116. doi: 10.26599/fmh.2025.9420116

[113] Zhang B Ye H Zhu XM Hu JN Li HY Tsao R . Esterification enhanced intestinal absorption of ginsenoside Rh2 in Caco-2 cells without impacts on its protective effects against H₂O₂-induced cell injury in human umbilical vein endothelial cells (Huvecs). J Agric Food Chem. (2014) 62:2096–103. doi: 10.1021/jf404738s, 24524563

[114] Tang X Liu C Wang B Zhang M Mao B Zhang Q . Enhancing the anti-fatigue effect of ginsenoside extract by *Bifidobacterium animalis* subsp. *lactis* CCFM1274 fermentation through biotransformation of Rb1, Rb2 and Rc to Rd. Food Biosci. (2024) 62:105251. doi: 10.1016/j.fbio.2024.105251

[115] Zhang X Chen S Duan F Liu A Li S Zhong W . Prebiotics enhance the biotransformation and bioavailability of ginsenosides in rats by modulating gut microbiota. J Ginseng Res. (2021) 45:334–43. doi: 10.1016/j.jgr.2020.08.001, 33841014 PMC8020290

[116] Yue X Guo H Wang G Li J Zhai Z Wang Z . A tailored phytosomes based nose-to-brain drug delivery strategy: silver bullet for Alzheimer's disease. Bioact Mater. (2025) 44:97–115. doi: 10.1016/j.bioactmat.2024.09.039

[117] Kadriya A Falah M. Nanoscale Phytosomes as an emerging modality for Cancer therapy. Cells. (2023) 12:1999. doi: 10.3390/cells12151999, 37566078 PMC10417745

[118] Li J Zheng M Shimoni O Banks WA Bush AI Gamble JR . Development of novel therapeutics targeting the blood-brain barrier: from barrier to carrier. Adv Sci. (2021) 8:e2101090. doi: 10.1002/advs.202101090 PMC8373165 34085418

[119] Kim YJ Perumalsamy H Markus J Balusamy SR Wang C Ho Kang S . Development of *Lactobacillus kimchicus* DCY51(T)-mediated gold nanoparticles for delivery of ginsenoside compound K: in vitro photothermal effects and apoptosis detection in cancer cells. Artif Cells Nanomed Biotechnol. (2019) 47:30–44. doi: 10.1080/21691401.2018.1541900, 30663395

[120] Chellappan DK Yee NJ Kaur Ambar Jeet Singh BJ Panneerselvam J Madheswaran T Chellian J . Formulation and characterization of glibenclamide and quercetin-loaded chitosan nanogels targeting skin permeation. Ther Deliv. (2019) 10:281–93. doi: 10.4155/tde-2019-0019, 31094299

[121] Mi XJ Choi HS Park HR Kim YJ. Structural characterization and anti-inflammatory properties of green synthesized chitosan/compound K-gold nanoparticles. Int J Biol Macromol. (2022) 213:247–58. doi: 10.1016/j.ijbiomac.2022.05.177, 35640850

[122] Xu XH Yuan TJ Dad HA Shi MY Huang YY Jiang ZH . Plant exosomes as novel Nanoplatforms for Microrna transfer stimulate neural differentiation of stem cells *in vitro* and *in vivo*. Nano Lett. (2021) 21:8151–9. doi: 10.1021/acs.nanolett.1c02530, 34586821

[123] Wang M Yang Y Guo Y Tan R Sheng Y Chui H . Xiaoxuming decoction cutting formula reduces LPS-stimulated inflammation in BV-2 cells by regulating miR-9-5p in microglia exosomes. Front Pharmacol. (2023) 14:1183612. doi: 10.3389/fphar.2023.1183612 37266151 PMC10229826

[124] Imtiaz C Kim C Paeng D-G. Focused ultrasound-mediated blood-brain barrier opening enhances delivery of Rg3 ginseng nanoparticles in a Parkinson's disease mouse model. Int J Pharm X. (2026) 12:100583. doi: 10.1016/j.ijpx.2026.100583, 42370027 PMC13293653

[125] Jo Y Kim S Jeong J Lee HJ. Ultrasound brain stimulation technologies for targeted therapeutics. Nat Electron. (2025) 8:647–62. doi: 10.1038/s41928-025-01420-3

[126] Rezai AR Teixeira CVL Ranjan M Adams G Arsiwala T Finomore V . Exploring low-intensity focused-ultrasound neuromodulation in Alzheimer's disease. Alzheimers Dement. (2025) 21:e100580. doi: 10.1002/alz70858_100580

[127] Hu K Ye J Fan P Zheng R Wang S Peng Y . Targeting and reprogramming microglial phagocytosis of neutrophils by ginsenoside Rg1 nanovesicles promotes stroke recovery. Bioact Mater. (2025) 47:181–97. doi: 10.1016/j.bioactmat.2025.01.017, 39906647 PMC11790502

[128] Kim B Kim YS Li W Kwon E-B Chung H-S Go Y . Ginsenoside Rg5, a potent agonist of Nrf2, inhibits HSV-1 infection-induced neuroinflammation by inhibiting oxidative stress and Nf-κB activation. J Ginseng Res. (2024) 48:384–94. doi: 10.1016/j.jgr.2024.01.006, 39036736 PMC11258381

[129] Quan Q Li X Feng J Hou J Li M Zhang B. Ginsenoside Rg1 reduces β-amyloid levels by inhibiting cdκ5-induced Pparγ phosphorylation in a neuron model of Alzheimer's disease. Mol Med Rep. (2020) 22:3277–88. doi: 10.3892/mmr.2020.11424, 32945455 PMC7453505

