# Supplementary material for: A recent review on ginseng’s effects on neurodegenerative diseases: active ingredients, mechanisms of action, applications, and delivery strategies
Source: Front Nutr. 2026 Aug 19;13:1912520. doi: 10.3389/fnut.2026.1912520 (PMC13536947; doi:10.3389/fnut.2026.1912520)
Supplement: Supplementary file 1 [file Table_1.docx]

Supplementary Table 1. Mechanism of action of ginseng and active ingredients to improve NDDs.

| **Active ingredients** | **Mechanism of action** | **Assays** | **Model** | **Dose** | **Main findings** | **References** |
| --- | --- | --- | --- | --- | --- | --- |
| Ginsenoside Rg3 (GRg3) | inhibit Aβ production and deposition | *in vitro* and *in vivo* | Aβ exposure in SH-SY5Y cells  Bilateral intracerebroventricular (ICV) injection of aggregating β-amyloid (Aβ1–42) into Wistar rats | 2.5 to 40 µM  10 mg/kg | PEGylated GRg3-loaded niosomes for the potential treatment of Alzheimer's disease. | (Barani et al., 2025) |
| Ginsenoside Re (G-Re) | via SIRT1/AMPK/mTOR signaling | *in vitro* and *in vivo* | J774A.1 murine macrophages exposed to Aβ1-42 and HT22 mouse hippocampal Cells deprived of Serum  Aβ1-42 mouse model | 2.5, 5, or 7.5 μg/mL  5 mg/kg | G-Re reduces mtROS levels, maintains mitochondrial membrane potential, and activates autophagy via the SIRT1/AMPK/mTOR signaling pathway, thereby inhibiting inflammasome activation. | (Madhi et al., 2026) |
| Ginsenoside Rg2 (GRg2) | regulation of gut microbiota and metabolites, anti-neuroinflammatory effects | *in vivo* | APP/PS1 mice | 50 mg/kg | GRg2 effectively inhibits the growth of Bacteroides and Helicobacter pylori, and also reduces the expression of neuroinflammatory mediators. | (Dilida et al., 2025) |
| Ginsenoside C-K (GCK) | inhibited NF-κB pathway | *in vitro* | BV2 and HT22 cells | 0–10 μM | GCK directly inhibited the neurotoxicity of Aβ and indirectly regulated the homeostasis of microglia. | (Xie et al., 2025) |
| Ginsenoside SumI | mitigated protein aggregation by activating the autophagy-lysosome pathway | *in vivo* | Caenorhabditis elegans | 13.8 mM | SumI effectively lowered the pH of lysosomes, promoted the formation of autophagosomes, and facilitated the transport of misfolded proteins to lysosomes for degradation in Caenorhabditis elegans. | (Zhao et al., 2025) |
| Ginsenoside Rg1 | downregulated FGR levels | *in vivo* | C57BL/6 J mice were injected with Aβ1–42 in the right lateral ventricle | 40 mg/kg | Rg1 treated Alzheimer's disease by targeting FGR and downregulating its expression. | (Quan et al., 2025) |
| Ginsenoside Ro | through the modulation of the IBA1/GFAP-MAPK pathway | *in vivo* | APP/PS1 mice | 15 mg/kg or 5 mg/kg | It reduced neuroinflammation by lowering pro-inflammatory cytokines, and simultaneously enhancing the anti-inflammatory cytokine IL-10. | (Li et al., 2025) |
| forest-grown ginseng (GSF) | Reduce neuroinflammation | *in vitro* | PC12 cells | 200 μg/mL, 100 μg/mL, 50 μg/mL | GSF alleviates neuroinflammation and alleviates Alzheimer's disease by regulating metabolic disturbances in amino acids, sphingolipids, unsaturated fatty acids, and arachidonic acid in mouse serum and brain tissue. | (Niu et al., 2025) |
| Ginsenoside Rg1 (Rg1) | regulated mitochondrial fission mediated by the AMPK/Drp1 signaling pathway | *in vitro* and *in vivo* | APP/PS1 mice  HT22 cells | 5 mg/kg/d or 10 mg/kg/d  1 μM, 5 μM, 10 μM | Rg1 regulates mitochondrial dynamics via the AMPK/Drp1 signaling pathway, thereby reducing synaptic and mitochondrial dysfunction in APP/PS1 mice and AD cell models. | (Zhang et al., 2025a) |
| Ginsenoside Rg1 (Rg1) | regulated the GATA4/PDE4A/PI3K/AKT axis | *in vitro* | Microglial cells (BV2) were treated with Aβ1-42 | 0, 0.5, 1, 10 μM | Rg1 alleviates Aβ1-42-induced apoptosis and inflammation in Alzheimer's disease-associated microglia via the GATA4/PDE4A/PI3K/AKT axis. | (Fang et al., 2025) |
| Ginsenoside Rg2 | inhibit the TLR4/MyD88/MMP9 inflammatory pathway | *in vitro* and *in vivo* | Co-culture system of MA-c and bEnd3  AlCl3 plus d-galactose (D-gal) caused AD mice | 20 μM  10 mg kg-1 and 20 mg kg-1 | G-Rg2 reduces the secretion of inflammatory factors and MMPs by inhibiting the activation of MA-c and the interaction between TLR4 and MyD88, thereby maintaining the stability of TJs in bEnd3. | (Lu et al., 2024) |
| Ginsenoside Rg2 | regulation of the lysosomal pathway | *in vivo* | utilized scopolamine to induce memory impairment in mice | 10, 20, and 30 mg kg^–1^ | Rg2 primarily improved AD mice by downregulating the expression of LGMN, LAMP1, and PSAP proteins through the regulation of the lysosomal pathway. | (Yin et al., 2024) |
| Total ginsenosides (TG) | reduced the production of Aβ | *in vitro* and *in vivo* | N2a/APP695 cells  APP/PS1 mice | 100 mg/L  80 mg/kg and 160 mg/kg | TG treatment reduced the expression of the genes BACE1, PS1, and PS2, which are associated with the amyloid precursor protein pathway. | (He et al., 2024) |
| Ginsenosides Rg1 and Rg2 | Lysosomal hydrolases are activated, and the enhanced autophagy further promotes the clearance of Aβ (1-42) | *in vitro* | SH-SY5Y cells | 50 μM | Rg1 and Rg2 upregulates autophagy in neurons, leading to increased levels of LC3II and Beclin-1 proteins and a decrease in the number of p62 particles. | (Liu et al., 2024b) |
| Ginsenoside Rg5 | inhibited Nrf2 pathway | *in vitro* | Vero and SK-N-SH cells | 5 or 10 μM | Rg5 treatment inhibits ROS/NF-κB signaling via NRF2 induction and reduces inflammatory cytokines. | (Kim et al., 2024) |
| Ginsenoside Rg1 | promotes β‑amyloid peptide degradation through inhibition of the ERK/PPARγ phosphorylation pathway | *in vitro* | Rat hippocampal neuron | 8 µM | Rg1 exerts a neuroprotective effect against AD by inhibiting the ERK/PPARγ phosphorylation pathway, thereby increasing IDE expression, promoting Aβ degradation, and reducing neuronal apoptosis. | (Quan et al., 2024) |
| Ginsenoside RK3 | through the CREB/BDNF signaling pathway | *in vivo* | APP/PS1 mice | 1 or 10 mg/kg | RK3 promotes neurogenesis and synapse formation via the CREB/BDNF signaling pathway, thereby enhancing learning and cognitive abilities in APP/PS1 mice. | (She et al., 2024) |
| Ginseng protein (GP) | reduced the aggregation of α - Syn | *in vivo* | 1-methyl-4-phenyl-1,2,3,6-tetrahydropyridine (MPTP)-induced PD mice | 16, 32 or 48 mg/kg | GP can improve behavioral deficits, brain and intestinal pathological damage, and gut microbiota dysbiosis in mice with PD. | (Sun et al., 2026b) |
| CK | α-Syn-specific aggregation were suppressed | *in vivo* | MPTP-induced PD mouse model | 10 or 40 mg/kg | By correlating behavioral, physiological, and biochemical markers with the gut microbiota, the WB study elucidates the neuroprotective effects of CK in PD mice | (Yang et al., 2025) |
| Ginsenoside Rk3 | via intestinal microbial homeostasis remodeling | *in vivo* | rotenone-induced PD mouse model | 80 mg/kg | Rk3 slows the progression of PD by reducing neuroinflammation through a mechanism independent of the gut microbiota. | (Zhou et al., 2025) |
| Ginsenoside Re | the restoration of gut microbiota ingredient | *in vivo* | MPTP-induced PD mouse model | 10 or 40 mg/kg | The neuroprotective effects of ginsenoside Re in PD mice may be associated with the gut microbiota and serum metabolism. | (Li et al., 2026a) |
| Ginsenoside Re | through the Drp1-Atg1/ULK1 axis | *in vivo* | Drp1L94A knock-in (KI) mice | 10 mg/kg | Re Restores Mitochondrial Homeostasis via Atg1/ULK1-Dependent Fission-Mitochondria Fusion in Age-Related Degeneration. | (Jin et al., 2025) |
| Purified panaxadiol saponin fraction (PDSF) | inhibiting NF-κB p65 in CEC | *in vivo* | rotenone (ROT)-induced PD | 20, 40 and 60 mg/kg | PDSF exerts a significant protective effect against ROT-induced Parkinson’s disease in rats by protecting CEC, glial cells, and DN. | (Wang et al., 2024c) |
| R-PPD and S-PPD | protected MPP+-induced neuron injury and mitochondrial dysfunction | *in vivo* | MPTP-induced PD mouse model | 20 mg/kg, ig | They reduced the abnormal expression of Cyt C, Bax, caspase-3 and Bcl-2. | (Zhao et al., 2024) |
| Ginsenoside Rg3 | TRKA-GRB2-CRLS1 axis | *in vivo* | subacute MPTP-induced PD model | 5, 20 or 80 mg/kg | Rg3 exhibit promising potential for the prevention of PD by bolstering cardiolipin expression and reinstating mitochondrial homeostasis. | (Qi et al., 2024) |
| Ginsenoside Rg1 | alleviated obstacles in αSyn autophagic degradation | *in vivo* | Human αSyn A53T-overexpressing mice; RTP801 knockout (RTP801−/−) mice; C57BL/6J | 40 mg/ kg/d | Rg1 alleviates the impairment of α-Syn autophagic degradation by mitigating the RTP801-TXNIP-mediated deficiency of ATP13A2. | (Wang et al., 2025b) |
| Rg1 | Reduced Aβ levels | *in vitro* | Rat hippocampal neurons cells | 60 μM | Rg1 affects PPARγ target gene expression by down-regulating CDK5 expression. | (Quan et al., 2020) |
| Rb1 | Inhibition of Aβ and phosphorylated Tau protein production | *in vivo* | AlCl3 induces AD | 70 mg/kg/d | Rb1 treatment improved passive avoidance memory deficits, attenuated the accumulation of Aβ and phosphorylated tau proteins, and attenuated histopathological changes in the cerebral cortex of AlCl3-administered rats. | (Shalaby et al., 2023) |
| Rg1 | Reduced Aβ deposition | *in vivo/ in vitro* | 5XFAD mouse model /SH-SY5Y cells | 10 mg/kg/d | Induction of microglia phagocytosis and reduction of Aβ deposition in the hippocampus of AD mice. | (Wang et al., 2023b) |
| ginsenosides Rf and Rg3 | reduce mHtt aggregates | *in vivo* | neural stem cells were isolated from the R6/2 mouse brain | 1 μM | They upregulate or tend to upregulate molecules associated with mitochondrial biogenesis and downregulated apoptosis-related molecules. | (Lee et al., 2021) |
| ginsenoside (compound K, CK) | suppressed the activation of ATM/AMPK | *in vivo* | R6/2 transgenic mouse model of HD | 30 or 60 mg/kg | CK upregulates the expression of Bcl-2, which protects striatal cells from toxicity caused by mHtt and excessive AMPK activation. | (Hua et al., 2022) |
| Ginsenoside compound K (CK) | inhibiting the formation of toxic RNA foci | *in vitro* and *in vivo* | HEK-293T and Caenorhabditis elegans strains | 5、10、20、40、60、80、100 and 120 μM | CK preferentially interacts with G-tetramers formed by GGGGCC repeat sequences rather than complex polymers. | (Li et al., 2026b) |
| Gintonin | Reduced levels of the LPA1 receptor in the spinal cord | *in vivo* | G93A-SOD1 transgenic mouse | 50 or 100 mg/kg/day, p.o. | The improvement in ALS symptoms mediated by Gintonin may be related to the reduction of neuronal loss and oxidative stress through the regulation of the LPA1 receptor in the spinal cord. | (Nam et al., 2021) |
| ginsenosides Rg1 (G-Rg1) | suppressing the NF-κB/Bcl-2 signaling pathway | *in vitro* | SOD1G93A-NSC34 cells | 60 μg/mL | G-Rg1 reduced the apoptotic rate induced by betulinic acid (BetA), an agonist of the NF-κB signaling pathway. | (Huang et al., 2023) |
